# Supplementary material for: Community assessment of crustose calcifying red algae as coral recruitment substrates
Source: PLoS One. 2022 Jul 22;17(7):e0271438. doi: 10.1371/journal.pone.0271438 (PMC9307205; doi:10.1371/journal.pone.0271438)
Supplement: S2 Table — The GenBank accession numbers is provided for each gene. (DOCX) [file pone.0271438.s002.docx]

**S2 Table. Taxon list from the Peña et al. [69] sequence alignment utilized to phylogenetically place each Corallinales species identified in this study**. The GenBank accession numbers is provided for each gene.

|  |  |  | GenBank Accession Numbers | | | | | | |
| --- | --- | --- | --- | --- | --- | --- | --- | --- | --- |
| Taxon | **Order** | **Species** | **COI** | ***psbA*** | ***rbc*L** | **23S rRNA** | **SSU rRNA** | **LSU rRNA** | **EF2** |
| Taxon 001 | Corallinales | Lithophyllaceae sp. (voucher ID MAD2358, Madagascar) | MT325717 | MT325762 | ------------- | ------------- | ------------- | MT325700 | ------------ |
| Taxon 001a | Corallinales | Lithophyllaceae sp. (voucher ID VPF00502A, Atlantic Spain) | MT325711 | MT325756 | MT325734 | ------------- | ------------- | MT325687 | ------------ |
| Taxon 002 | Corallinales | *Metagoniolithon radiatum* | ------------- | GQ917496 | ------------- | ------------- | GQ917432 | GQ917369 | ------------ |
| Taxon 002b | Corallinales | *Metagoniolithon stelliferum* | ------------- | GQ917497 | ------------- | ------------- | GQ917433 | GQ917370 | ------------ |
| Taxon 003 | Corallinales | Lithophyllaceae sp.(voucher ID MAD1024B, Madagascar) | MT325705 | MT325748 | ------------- | ------------- | ------------- | MT325678 | ------------ |
| Taxon 003a | Corallinales | Lithophyllaceae sp. (voucher ID MAD938B, Madagascar) | MT325709 | MT325754 | ------------- | ------------- | MT325665 | MT325685 | ------------ |
| Taxon 004 | Corallinales | *Dawsoniolithon* sp. (as Uncultured Corallinales, Bittner et al. 2011) | GQ917265 | GQ917454 | ------------- | ------------- | GQ917399 | GQ917328 | ------------ |
| Taxon 005 | Corallinales | *Dawsoniolithon conicum* (as *Pneophyllum conicum*, Bittner et al. 2011) | GQ917272 | GQ917464 | ------------- | HQ423076 | GQ917408 | GQ917337 | ------------ |
| Taxon 006 | Corallinales | *Harveylithon* sp. (as Uncultured Corallinales, voucher ID LBC0600, Bittner et al. 2011) | GQ917264 | GQ917453 | MT325719 | ------------- | GQ917398 | GQ917327 | ------------ |
| Taxon 007 | Corallinales | *Harveylithon* sp. (voucher ID MAS304_1C, Oman) | MT325714 | MT325758 | ------------- | ------------- | ------------- | MT325693 | ------------ |
| Taxon 008 | Corallinales | *Porolithon* sp. (as *Hydrolithon onkodes*, voucher ID LBC0820_P3, Bittner et al. 2011) | GQ917291 | GQ917483 | MT325738 | KM073308 | GQ917373 | GQ917357 | ------------ |
| Taxon 009 | Corallinales | *Porolithon* sp. (as *Hydrolithon* sp., Bittner et al. 2011) | GQ917286 | GQ917478 | ------------- | HQ42271 | GQ917422 | GQ917352 | ------------ |
| Taxon 010 | Corallinales | *Porolithon* *onkodes* (as *Hydrolithon* sp., voucher ID LBC0678, Bittner et al. 2011) | GQ917276 | GQ917469 | ------------- | ------------- | GQ917412 | GQ917341 | ------------ |
| Taxon 011 | Corallinales | *Porolithon* sp. (as *Hydrolithon* sp., Bittner et al. 2011) | GQ917287 | GQ917479 | ------------- | ------------- | GQ917423 | GQ917353 | ------------ |
| Taxon 012 | Corallinales | *Porolithon* sp.(as *Hydrolithon* sp., voucher ID LBC0882, Bittner et al. 2011) | GQ917301 | GQ917493 | MT325720 | ------------- | GQ917429 | GQ917366 | ------------ |
| Taxon 013 | Corallinales | Lithophyllaceae sp. (voucher ID MAD2344, Madagascar) | MT325715 | MT325759 | MT325737 | ------------- | ------------- | MT325694 | ------------ |
| Taxon 014 | Corallinales | Lithophyllaceae sp. (voucher ID NVT1054B, Vietnam) | MT325704 | MT325747 | MT325722 | ------------- | MT325662 | MT325677 | ------------ |
| Taxon 015 | Corallinales | *Adeylithon bosencei* (as Hydrolithon sp., voucher ID LBC0720, Bittner et al. 2011) | GQ917284 | GQ917476 | MT325731 | ------------- | GQ917420 | GQ917349 | ------------ |
| Taxon 016 | Corallinales | *Hydrolithon* cf. *boergesenii* | GQ917257 | GQ917447 | ------------- | ------------- | GQ917378 | GQ917321 | ------------ |
| Taxon 017 | Corallinales | *Hydrolithon reinboldii* | GQ917293 | GQ917485 | ------------- | HQ423071 | GQ917376 | GQ917359 | ------------ |
| Taxon 019 | Corallinales | *Chamberlainium* sp. (voucher ID LLG4403B, Australia) | ------------- | MT325753 | ------------- | ------------- | ------------- | MT325684 | ------------ |
| Taxon 020 | Corallinales | *Spongites hyperellus* | ------------- | GQ917495 | ------------- | ------------- | GQ917431 | GQ917368 | ------------ |
| Taxon 021 | Corallinales | *Pneophyllum fragile* | ------------- | KT783426 | ------------- | ------------- | ------------- | ------------- | ------------ |
| Taxon 022 | Corallinales | *Pneophyllum cetinaensis* (voucher ID PC0145164, Žuljević et al. 2016) | ------------- | KT783433 | ------------- | ------------- | ------------- | MT325690 | ------------ |
| Taxon 023 | Corallinales | *Spongites yendoi* | ------------- | DQ167907 | KT184848 | ------------- | EF628234 | ------------- | ------------ |
| Taxon 024 | Corallinales | Lithophyllaceae sp. (as Uncultured Corallinales, voucher ID LBC0707, Bittner et al. 2011) | GQ917589 | GQ917960 | MT325725 | ------------- | MT325664 | MT325682 | ------------ |
| Taxon 025 | Corallinales | *Lithophyllum* sp. (voucher LBC0714, Bittner et al. 2011) | GQ917282 | GQ917474 | MT325735 |  | GQ917418 | GQ917347 | ------------ |
| Taxon 026 | Corallinales | *Lithophyllum hibernicum* | GQ917250 | GQ917440 | KR708590 | KM073316 | GQ917385 | GQ917313 | ------------ |
| Taxon 027 | Corallinales | *Lithophyllum byssoides* (voucher ID VPF00306, Atlantic Spain) | MT325702 | MT325743 | KR708574 | ------------- | JQ896251 | MT325673 | ------------ |
| Taxon 028 | Corallinales | *Lithophyllum* sp. (voucher ID MAD0081, Madagascar) | MT325707 | MT325750 | ------------- | ------------- | JQ896235 | MT325680 | ------------ |
| Taxon 029 | Corallinales | *Lithophyllum* sp. (voucher ID LBC0946, Bittner et al. 2011) | GQ917691 | GQ918126 | MT325732 | HQ421552 | MT325667 | HQ422479 | ------------ |
| Taxon 031 | Corallinales | *Tinanoderma* sp. | KJ418416 | KJ418413 | KJ652016 | ------------- | ------------- | KJ412335 | ------------ |
| Taxon 032 | Corallinales | *Lithophyllum pustulatum* (voucher ID VPF00095, Atl. Spain) | MT325701 | MT325742 | KM369168 | KM073327 | KM073290 | MT325672 | ------------ |
| Taxon 033 | Corallinales | *Lithothrix aspergillum* | JQ615866 | JQ422237 | HQ322336 | ------------- | ------------- | ------------- | JQ422275 |
| Taxon 034 | Corallinales | *Lithophyllum kotschyanum* |  | AB576029 | KX020467 | KM073321 | AB576008 | KM977984 | ------------ |
| Taxon 035 | Corallinales | *Tinanoderma* sp. | GQ917285 | GQ917477 | ------------- | ------------- | GQ917421 | GQ917350 | ------------ |
| Taxon 036 | Corallinales | *Lithophyllum* sp. (voucher ID LBC0680, Bittner et al. 2011) | GQ917277 | GQ917470 | MT325723 | ------------- | GQ917413 | GQ917342 | ------------ |
| Taxon 037 | Corallinales | *Amphiroa* sp. (voucher ID LBC0865, Bittner et al. 2011) | GQ917299 | GQ917491 | MT325726 | ------------- | GQ917428 | GQ917364 | ------------ |
| Taxon 038 | Corallinales | *Amphiroa* sp. | GQ917246 | GQ917435 | ------------- | ------------- | GQ917380 | GQ917308 | ------------ |
| Taxon 039 | Corallinales | *Amphiroa* sp. (voucher ID LBC0708, Bittner et al. 2011) | GQ917280 | GQ917472 | MT325728 | ------------- | GQ917416 | GQ917345 | ------------ |
| Taxon 040 | Corallinales | *Amphiroa fragilissima* | GQ917303 | GQ917498 | U04039 | KM044012 | KY987580 | EF033599 | ------------ |
| Taxon 054 | Corallinales | *Mastophora/Lithoporella* | GQ917260 | GQ917449 | ------------- | ------------- | GQ917394 | GQ917323 | ------------ |
| Taxon 055 | Corallinales | *Jania longifurca* | GQ917251 | GQ917441 | KM369140 | ------------- | GQ917386 | GQ917314 | ------------ |
| Taxon 056 | Corallinales | *Jania* sp. | GQ917514 | GQ917712 | ------------- | HQ421368 | ------------- | HQ422166 | ------------ |
| Taxon 057 | Corallinales | *Jania rubens* (voucher ID VPF00439, Atlantic Spain) | MT325713 | MK308537 | KM044024 | KM044014 | KM044029 | MT325691 | ------------ |
| Taxon 058 | Corallinales | *Jania sagittata* | JQ615844 | JQ422232 | KC134331 | ------------- | KM369032 | KC157591 | KC130175 |
| Taxon 069 | Corallinales | *Mastophora rosea* (voucher ID LBC0866, Bittner et al. 2011) | GQ917300 | GQ917492 | MT325729 | ------------- | MT325666 | GQ917365 | ------------ |
| Taxon 070 | Corallinales | *Mastophora pacifica* | GQ917302 | GQ917494 | KM369152 | HQ420920 | GQ917430 | GQ917367 | ------------ |
| Taxon 081 | Corallinales | *Spongites* sp. (voucher ID LLG2579, Rösler et al. 2016) | KP682496 | MT325752 | ------------- | ------------- | ------------- | MT325683 | ------------ |
| Taxon 082 | Corallinales | *Spongites fruticulosus* (voucher ID VPF00027, Rösler et al. 2016) | MT325710 | MT325755 | ------------- | KM073335 | KM073306 | MT325686 | ------------ |
| Taxon 083 | Corallinales | *Arthrocardia corymbosa* | ------------- | JQ917408 | JN701475 | HQ421500 | ------------- | ------------- | ------------ |
| Taxon 084 | Corallinales | *Chiharaea bodegensis* | HM918942 | JQ677011 | JQ677000 | ------------- | KC157576 | KC157588 | KC130170 |
| Taxon 085 | Corallinales | *Calliarthron cheilosporioides* | JQ615594 | JQ422199 | HQ322299 | ------------- | CTU60944 | ------------- | JQ422270 |
| Taxon 086 | Corallinales | *Ellisolandia elongata* | JQ615843 | JQ422231 | JX315327 | ------------- | FM180099 | ------------- | JQ422258 |
| Taxon 087 | Corallinales | *Corallina caespitosa* | GQ917248 | GQ917438 | JQ615683 | KC478072 | GQ917383 | GQ917311 | KC130168 |
| Taxon 088 | Corallinales | *Neogoniolithon* sp. (voucher ID FRA1402, Guadeloupe, West Indies) | KP682495 | KP682501 | ------------- | ------------- | MT325668 | MT325692 | ------------ |
| Taxon 089 | Corallinales | *Neogoniolithon* sp. (voucher ID LBC0584, Bittner et al. 2011) | GQ917262 | GQ917451 | MT325730 | ------------- | GQ917396 | GQ917325 | ------------ |
| Taxon 090 | Corallinales | *Neogoniolithon* sp. | GQ917290 | GQ917482 | ------------- | ------------- | GQ917424 | GQ917356 | ------------ |
| Taxon 091 | Corallinales | *Neogoniolithon* sp. (voucher ID LBC0843, Bittner et al. 2011) | GQ917297 | GQ917489 | MT325727 |  | GQ917434 | GQ917362 | ------------ |
| Taxon 092 | Corallinales | *Neogoniolithon brassica-florida* (voucher ID VPF00284, Med. France) | KM392368 | MT325745 | ------------- | HQ422477 | JQ896257 | MT325675 | ------------ |
| Taxon 093 | Corallinales | *Neogoniolithon* sp. (voucher ID LBC0433, Bittner et al. 2011) | GQ917253 | GQ917442 | MT325733 | ------------- | GQ917388 | GQ917316 | ------------ |
| Taxon 094 | Corallinales | *Neogoniolithon* sp. (as Uncultured Corallinales, Bitnner et al. 2001) | GQ917274 | GQ917466 | ------------- | ------------- | GQ917410 | GQ917339 | ------------ |
| Taxon 095 | Corallinales | *Amphiroa valonioides* | HQ422698 | ------------- | ------------- | HQ421023 | ------------- | HQ422411 | ------------ |
| Taxon 096 | Corallinales | *Amphiroa foliacea* | HQ422626 | ------------- | ------------- | HQ420962 | ------------- | HQ421910 | ------------ |
| Taxon 097 | Corallinales | *Lithophyllum* sp. | HQ422960 | ------------- | ------------- | ------------- | ------------- | HQ422022 | ------------ |
| Taxon 098 | Corallinales | *Lithophyllum insipidum* | HQ423068 | ------------- | ------------- | HQ421545 | ------------- | HQ422473 | ------------ |
| Taxon 102 | Corallinales | *Jania* sp. | HQ423038 | ------------- | ------------- | HQ421370 | ------------- | HQ422269 | ------------ |
| Taxon 103 | Corallinales | *Jania* sp. | HQ422629 | ------------- | ------------- | HQ421458 | ------------- | HQ421768 | ------------ |
| Taxon 106 | Corallinales | *Spongites* sp. | HQ422715 | ------------- | ------------- | HQ420977 | ------------- | HQ421807 | ------------ |
| Taxon 107 | Corallinales | *Porolithon gardineri* | HQ423069 | ------------- | ------------- | HQ421546 | ------------- | HQ422474 | ------------ |
| Taxon_131 | Corallinales | *Pneophyllum coronatum* | ------------- | DQ168008 | ------------- | ------------- | KM369048 | ------------- | ------------ |
| Taxon_132 | Corallinales | *Pneophyllum fragile* | ------------- | FJ361387 | KM369155 | ------------- | KM369043 | ------------- | ------------ |
| Taxon_133 | Corallinales | *Metamastophora flabellata* | ------------- | ------------- | ------------- | ------------- | AY234240 | ------------- | ------------ |
| Taxon_134 | Corallinales | *Metamastophora flabellata* | ------------- | ------------- | ------------- | ------------- | AY234239 | ------------- | ------------ |
| Taxon_135 | Corallinales | *Mastophora* sp. | ------------- | ------------- | ------------- | ------------- | MG826386 | MG821596 | ------------ |
| Taxon_136 | Corallinales | *Parvicellularium leonardii* | ------------- | MG851066 | ------------- | ------------- | MG826383 | MG821592 | ------------ |
| Taxon_137 | Corallinales | *Parvicellularium* sp. | ------------- | MG851074 | ------------- | ------------- | ------------- | MG821601 | ------------ |
| Taxon_138 | Corallinales | *Porolithon* cf. *craspedium* | ------------- | MG851057 | ------------- | ------------- | MG826374 | MG821582 | ------------ |
| Taxon_139 | Corallinales | *Spongites* sp. | MG851045 | MG851062 | ------------- | ------------- | MG826379 | MG821588 | ------------ |
| Taxon_140 | Corallinales | *Spongites* sp. | MG851047 | MG851073 | ------------- | ------------- | MG826390 | MG821600 | ------------ |
| Taxon_141 | Corallinales | *Neogoniolithon* sp. | MG851044 | MG851061 | ------------- | ------------- | MG826378 | MG821586 | ------------ |
| Taxon_142 | Corallinales | *Chamberlainium* sp. | ------------- | MG851078 | ------------- | ------------- | MG826394 | ------------- | ------------ |
| Taxon_143 | Corallinales | *Harveylithon munitum* | ------------- | KM407531 | ------------- | ------------- | ------------- | KM073336 | ------------ |
| Taxon_144 | Corallinales | *Harveylithon rupestre* | ------------- | KM407535 | ------------- | ------------- | KM073303 | ------------- | ------------ |
