# Supplementary material for: Community assessment of crustose calcifying red algae as coral recruitment substrates
Source: PLoS One. 2022 Jul 22;17(7):e0271438. doi: 10.1371/journal.pone.0271438 (PMC9307205; doi:10.1371/journal.pone.0271438)
Supplement: S3 Table — Bold taxa names are generitypes. (DOCX) [file pone.0271438.s003.docx]

**S3 Table. GenBank accession numbers or BOLD Systems identification numbers (*) of COI-5P sequences of Peyssonneliales taxa used in Fig 3**. Bold taxa names are generitypes.

| **Taxa** | **Specimen Voucher** | **GenBank/BOLD** |
| --- | --- | --- |
| ***Ramicrusta nanhaiensis*** | GWS002520 | JX969713 |
| ***Polystrata* cf. *dura*** | VT097 | ABMMC1983-07* |
| ***Polystrata* cf. *dura*** | VT159 | ABMMC1984-07* |
| *Metapeyssonnelia* sp. | 6552 | ABMMC320-06* |
| *Metapeyssonnelia* sp. | 6558 | ABMMC323-06* |
| *Metapeyssonnelia* sp. | VT086 | ABMMC1862-07* |
| ***Riquetophycus polypus*** | HSY-2014a | KJ398161 |
| *Sonderophycus fervens* | VT061 | JX969700 |
| *Cruoriella* sp. | VT166 | ABMMC1861-07* |
| ***Peyssonnelia squamaria*** | GWS018179 | JX969741 |
| ***Incendia crenata*** | VT095 | JX969714 |
| ***Bonnemaisonia asparagoides*** | GWS040634 | MN184297 |
