# Supplementary material for: Community assessment of crustose calcifying red algae as coral recruitment substrates
Source: PLoS One. 2022 Jul 22;17(7):e0271438. doi: 10.1371/journal.pone.0271438 (PMC9307205; doi:10.1371/journal.pone.0271438)
Supplement: S4 Table — Only substrate categories with coral recruits are listed. The first column lists the substrate categories with associated coral recruits. The second column shows the average precent cover of a substrate category on all tiles. The third column represents the total number of coral recruits associated with a substrate category. The last column shows the probability value of larval recruitment preference. (DOCX) [file pone.0271438.s004.docx]

**S4 Table. G-test for goodness of fit of recruitment preference to substrate categories.** Only substrate categories with coral recruits are listed. The first column lists the substrate categories with associated coral recruits. The second column shows the average precent cover of a substrate category on all tiles. The third column represents the total number of coral recruits associated with a substrate category. The last column shows the probability value of larval recruitment preference.

| **Substrate Category** | **Percent Cover** | **Coral Recruits** | ***P-*value** |
| --- | --- | --- | --- |
| Corallinales | 31.9% | 3 | <0.0001 |
| Lithophylloideae sp. 1 | 41.5% | 45 | <0.0001 |
| Lithophylloideae spp. 2-4 | 2.9% | 5 | 0.037 |
| Peyssonneliales spp. 4,7 | 6.8% | 4 | 0.977 |
| *Polystrata* spp. 1-3 | 3.8% | 3 | 0.650 |
