# Supplementary material for: Community assessment of crustose calcifying red algae as coral recruitment substrates
Source: PLoS One. 2022 Jul 22;17(7):e0271438. doi: 10.1371/journal.pone.0271438 (PMC9307205; doi:10.1371/journal.pone.0271438)
Supplement: Attachment — Submitted filename: 02_Response_to_reviewers.docx [file pone.0271438.s009.docx]

9^th^ March, 2022

Dear PLoS ONE Editors,

We thank the editor and the two reviewers for taking the time to provide generous comments on the manuscript, PONE-D-21-27555. Below is our response to each point raised by the academic editor and reviewers. We hope the manuscript has been edited to satisfyingly address their concerns and the manuscript will now be suited for publication.

Sincerely,

On behalf of all authors,

Mari E. Deinhart

**Reviewer #1**

**Specific comments:**

**Reviewer #1: Main Comments:
This is an interesting study that utilises modern molecular methods to examine diversity of crustose red algae and associated coral (Acropora surculosa) settlement patterns. However, there are parts of this manuscript that need significant revisions to improve the clarity, provide additional details on the methods and results of this study.
The use of these molecular methods to confirm identifications used in this study distinguishes this study from the increasing body of literature on coral settlement on coralline algae species. The large majority of previous studies are based on morphological identification which can be problematic due to the prevalence of cryptic species in coralline red algae. The authors should make a point of this throughout the manuscript.**

Thank you for your insight and bringing this to our attention. This revised manuscript addresses the comments and suggestions brought up by the reviewers. Notable sections have been reworded to improve clarity and provide more detail in the methods and results section. We made a point of bringing up how the molecular methods used in this study distinguish it from previous studies that morphologically identify coral larval preference to specific CCRA species for recruitment.

**Abstract
Pg2 ln17 – What does “…crustose coralline algae, CCA) are often used at the functional group level…” mean? Please clarify. Does this mean that coralline algae species are often lumped together in many studies?**

Thank you for asking for clarification. This sentence created confusion in the abstract, therefore, it was deleted from the abstract. The topic was introduced and further explained in the Introduction (line 109-113).

“Due to these identification challenges, CCRA are often grouped into a single functional group in monitoring and ecological surveys, which does not recognize the species richness of CCRA and the different ecological roles that CCRA species serve in marine environments, including tropical reefs, temperate reefs, and rhodolith beds [54].”

**Pg2 ln20- You should state that molecular methods were used to identify the species in the aims section of the abstract as this is a distinguishing point of this research.**

Thank you for point out that the original manuscript submission did not state molecular methods were used. We amended the abstract to state molecular methods were used to identify CCRA species which distinguishes it from previous studies identifying CCRA species for coral recruitment (line 23).

“In this study, DNA sequence analysis was used to identify CCRA species to (1) investigate the species richness and community composition of CCRA on experimental coral recruitment tiles and (2) assess if the coral *Acropora surculosa* preferred any of these CCRA species as recruitment substrates.”

**Pg2 ln22 – You haven’t said what any of the coralline species are. Perhaps reword to “…preference of the coral *Acropora surculosa* to any identified CCRA species.”**

Thank you, we have inserted that Lithophylloideae sp. 1 and Lithophylloideae sp. 2 were the preferred CCRA species for recruitment of *Acropora surculosa* larvae (line 29).

“Two closely related CCRA species, Lithophylloideae sp. 1 and Lithophylloideae sp. 2 (Corallinales), were significantly preferred as recruitment substrates over all other CCRA species.”

**Pg20 ln 24 – This sentence could be reduced to state that theses sequences do not match any in publicly available databases**

Thank you, we thought it would be best to rewrite the sentence as “The CCRA community assemblages on the coral recruitment tiles was species-rich, comprising 27 distinct CCRA species of the orders Corallinales and Peyssonneliales.” (line 28)

**Pg20 ln25-33 – This section of the abstract needs some rewording as it seems repetitive in parts and jumps back and forth between preferred settlement substrate and benthic cover on the tiles. I would avoid bring up the point that these other pacific corallines are likely assigned to the wrong genus of *Titanoderma* as this confuses the reader and isn’t relevant to the point you are trying to make. Perhaps just state that your two Lithophylloideae species are closely related to other coralline species in the pacific that have been shown to be preferred coral recruitment substrate.**

Thank you for pointing that out. The abstract was amended to avoid repetition and provide a clear and concise summary of the study. We provide further details about *Titanoderma* species being regarded as prime settlement substrates, however there is not valid reporting of the genus for Guam and Micronesia (line 33-36).

“*Titanoderma* species (Lithophylloideae, Corallinales) are typically regarded to be the prime settlement substrates for *Acropora* larvae in the tropical Pacific. The identification of *Titanoderma* taxa in recruitment studies in the Pacific region will be discussed in the context of our ecological and phylogenetic findings."

**Introduction
Pg3 ln 45 – What do you mean by “…that deposit calcium carbonate.” Are you talking about in the cell walls or into the ocean/sediment**

Thank you, this statement was reworded as CCRA being one of the main calcium carbonate producers and sequesters (line 53).

“CCRA are essential components of healthy reef systems because of their ecological importance as (1) some of the main calcium carbonate producers and carbon sequesters while creating benthic (micro)habitats [8],”

**Pg3 ln 50 – What is a suppressor of nutrient indicator algae?**

Thank you, line 55 now provide further detail explaining that CCRA can suppress seaweed (fleshy macroalgae) overgrowth.

“(4) suppressors of nuisance species overgrowth [11-13].”

**Pg5 ln 82-83 – Remove this final sentence of the paragraph. This isn’t relevant. If you want to retain this sentence you should be worded more broadly. i.e. *A. surculosa* is a popular study organism for the following reasons.**

Thank you, this sentence was removed and an explanation of why *A. surculosa* is used for restoration efforts on Guam is explained in lines 86-88.

“*A. surculosa* is a corymbose acroporid that has been well studied in Guam due to its common occurrence on forereefs, its fast growth rate, and its overall value as a reef builder [27,28].”

**Pg5 ln 97 – Perhaps cite Twist et al. 2020 as they discuss how caution must be taking with morpho-anatomical identification and the advantages of using molecular methods for coralline algae identification.
Twist, B. A., Cornwall, C. E., McCoy, S. J., Gabrielson, P. W., Martone, P. T., & Nelson, W. A. (2020). The need to employ reliable and reproducible species identifications in coralline algal research. Marine Ecology Progress Series, 654, 225-231**

Thank you, we agree that citing Twist et al. 2020 here would be beneficial (line 102).

**Pg6 ln 102 - Perhaps cite Hernandez-Kantun et al. 2016 and/or Maneveldt et al. 2017 as there is some evidence of wide species distributions with molecular identified corallines**
**Hernandez-Kantun, J. J., Gabrielson, P., Hughey, J. R., Pezzolesi, L., Rindi, F., Robinson, N. M., ... & Adey, W. (2016). Reassessment of branched Lithophyllum spp.(Corallinales, Rhodophyta) in the Caribbean Sea with global implications. Phycologia, 55(6), 619-639. sepecimens.
Maneveldt, G. W., Gabrielson, P. W., & Kangwe, J. (2017). Sporolithon indopacificum sp. nov.(Sporolithales, Rhodophyta) from tropical western Indian and western Pacific oceans: First report, confirmed by DNA sequence data, of a widely distributed species of Sporolithon. Phytotaxa, 326(2), 115-128.**

Thank you for recommending these papers. We have since cited both throughout this paragraph (lines 102-109).

**Pg7 ln103 – Steneck and Diether [53] is probably an inappropriate reference for stating that current species richness in this group is underestimated.**

Thanks for pointing out the inappropriate reference here. The citation of Steneck and Diether were deleted and instead, Ford et al. [54] was cited (lines 109-113).

“Due to these identification challenges, CCRA are often grouped into a single functional group in monitoring and ecological surveys, which does not recognize the species richness of CCRA and the different ecological roles that CCRA species serve in marine environments, including tropical reefs, temperate reefs, and rhodolith beds [54].”

**Pg7 ln 104 – Reword to “CCRA are generally lumped together as a single function group in monitoring and ecological surveys…” or something similar**

Thank you, we reworded this sentence in lines 113-117.

“Due to these challenges, CCRA are generally grouped together as a single functional groups in monitoring and ecological surveys, which does not recognize the species richness of CCRA and the different ecological roles that these CCRA serve in marine habitats, including tropical reefs, temperate reefs, and rhodolith beds [54].”

**Pg7 ln 105 – Not just their role in tropical reefs but also in temperate reef systems and rhodolith beds.**

Thank you, we added their roles in tropical reef, temperate reefs, and rhodolith beds (lines 116-117).

**Methods
Pg7 ln 121 – Please expand on the methods in this section to clearly describe how the coralline recruitment was recorded on the settlement plates. It is unclear how the tiles were covered in CCRA and if this was before the Coral settlement event or at the same time? How long were these tiles left for the CCRA to grow? How was settlement success of the coral recorded? Under a microscope? Using the photos? and after how many days? Is there a reason for using star shaped settlement tiles instead of circular or square ones?**

**Lines (134-172):**

Thank you for noting that this section needed to be expanded. We expanded this section (**Substrate categories on coral recruitment tiles**) by describing how long tiles were cured before coral spawning event and how the CCRA settled. A further description of how coral recruitments were identified in the photos and during extraction. This expanded paragraph in the materials and methods also explains why this particular shape of tiles were used in this study. (lines 130-168)

**Pg6 ln122-123 – This sentence doesn’t seem to be relevant or a good opening sentence for the methods. Perhaps start by explaining that star shaped tiles were left for X time to recruit CCRA and then state how A. surculose was settled in 2018. Then go onto explain how settlement success was recorded, that photos were taken for analysis and finally tissue sample was taken for DNA analysis for CCRA that A. surculose settled on along with 32 additional samples of distinct morphologies.**

We appreciate you indicated that more clarification in our methodology was necessary. We agree this first sentence was not relevant and was therefore deleted. A more in-depth explanation of the spawning and recruitment process is stated in this revision. Overall, the material and methods section was expanded to explain the methodology for recording recruitment, the image analysis, and the DNA extraction for CCRA (lines 138-168)

**Pg7 ln124 – Is there a reference for SECORE protocols? No need to state that the Raymundo Coral lab follows these protocols.**

SECORE is referenced and the statement about the Raymundo Coral lab is deleted (line 130).

“Twelve ceramic star-shaped coral recruitment tiles [**56**] were maintained in the flow-through seawater system at the University of Guam Marine Laboratory (UOGML) to recruit CCRA one month prior to the July 2018 *Acropora* spawning event.”

**Pg7 ln 133 – Were the tiles dried before DNA scrapping? If so, in air or silica gel?**

Great question! The samples for DNA extraction were taken from live tissue. The tiles were air dried after DNA extraction. We added an explanation in lines 151 (“At the same time, scrapings of live CCRA tissue samples were taken for DNA extraction.) & 156-158 (“Following DNA extraction, tiles were air-dried and deposited into the marine plant collection of the University of Guam Herbarium.”).

**Pg7 ln 146 – You don’t need to state that tissue samples were placed in 1.5ml epindorfs**

We deleted the 1.5ml epindorf statement.

**Pg8 ln153 – COI isn’t the official marker for red algae. Saunders suggested that we utilize a common marker (COI) for red algae to make results comparable across studies, but it is not the "official" barcode marker.**

Thank you for indicating the inaccuracy of that. This revision amended this sentence by explaining that it is a barcode marker that is regularly used for red algae (lines 190-192).

“The mitochondrial cytochrome c oxidase subunit I, COI-5P (roughly 664 bp), is a barcode marker that is regularly used for DNA barcoding of red algae [57] and was used for both the Corallinales and Peyssonneliales.”

**Pg8 ln 158 – Please clarify what markers were used for what CCRA – Was COI one was used for everything (including Peyssonneliales) and psbA and rbcL were used for Corallinales, Sporolithales & Hapalidiales (although it appears only specimens of the order Corallinales were found).**

Thank you for asking for a necessary clarification. This revision clarifies that the three markers (COI, *psb*A, and *rbc*L) were used for Corallinales, while COI was just used for Peyssonneliales (lines 192, 196, and 202).

“The mitochondrial cytochrome c oxidase subunit I, COI-5P (roughly 664 bp), is a barcode marker that is regularly used for DNA barcoding of red algae [57] and was used for both the Corallinales and Peyssonneliales.”

“Chloroplast photosystem II thylakoid membrane protein D1, *psb*A (roughly 950 bp), was used as a second barcode marker for the Corallinales.”

“The chloroplast ribulose-1, 5-biphosphate carboxylase large subunit, *rbc*L (roughly 1,350 bp), was amplified for a subset of Corallinales specimens from the coral recruitment tiles”

**Pg 8 ln 160 – Please include a citation for the statement that psbA is commonly used and has a high success rate. Perhaps Broom et al. 2008
Broom, J. E., Hart, D. R., Farr, T. J., Nelson, W. A., Neill, K. F., Harvey, A. S., & Woelkerling, W. J. (2008). Utility of psbA and nSSU for phylogenetic reconstruction in the Corallinales based on New Zealand taxa. Molecular phylogenetics and evolution, 46(3), 958-973.**

Thank you for this suggestion. We included Broom et al. in citation for *psb*A statement (line 199).

“P*sb*A is more conserved than COI-5P and is frequently used for CCRA identification because of its high success rate of amplification [**60**].”

**Pg8 ln 165 – What do you mean by the amplification profile?**

Thank you for asking for clarification. This statement was modified for clarification (lines 203-204).

“Amplification of *rbc*L used the primers F57 and rbcLrevNEW [62] following the PCR protocol outlined in Saunders and Moore [62].”

**Pg9 ln172 – A BLAST search searches the Genbank database**

Thank you, we changed the wording of for this sentence to clarify that BLAST search includes GenBank as well (lines 214-217).

“BLAST searches (Basic local alignment search tool) [64] of the consensus sequences were run to search for close matches in (1) a database of CCRA sequences from Guam and (2) online repositories such as GenBank and the Barcode of Life Database (BOLD) [65].”

**Pg9 ln 180 – Please clarify the ABGD settings used and on what platform this analysis was run**

Thank you, we cited the software used for the ABGD and indicated the species delimitation profile for each gene (lines 214-217).

“To assess species richness and to delimit putative species in this study, sequence divergence thresholds were calculated using the Automatic Barcode Gap Discovery (ABGD) [67] for COI-5P (3%), *psb*A (2.5%), and *rbc*L (0.9%) [57].”

**Pg10 ln 200 – It should be clarified earlier in the methods that a separate analysis was run for the Peyssonneliales**

Thank you, we agree it should be clarified that a separate analysis was run for Peyssonneliales phylogeny (line 213)**.**

**“**An alignment of COI-5P was made for the Peyssonneliales and a concatenated alignment of COI-5P, *psb*A, and *rbc*L for the Corallinales.”

**Pg10 ln 214- I suggest moving this section on substrate cover up to underneath the first section in the methods or even integrated with this first section to improve the flow and clarity.**

That you for that suggestion. This revision integrated the identifying recruitment preference with the first section (**Substrate categories on coral recruitment tiles**) in the methods (lines 159-178).

**Pg11 ln 215-16 – What do you mean by substrate categories? Are these CCRA morphological/ putative species groups? And does this include things such as bare settlement plate. Please reword to clarify**

Thank you, we agree that this needed more clarification in the Materials & Methods. Lines 159-163 were rewritten as “For the recruitment preference analysis based on cover data, CCRA taxa) were grouped into eight substrate categories that could be discerned visually and included one abiotic category (Table 1). The eight substrate categories included CCRA thalli that were not molecularly but were morphological similar to the CCRA species validated through DNA sequence analysis.”

**Pg12 ln230 – Table 1 description- there is no “species count column”**

Thank you, we have reworded column label and caption for Table 1.

“**Table 1. Description of the nine substrate categories found on the recruitment tiles.** N Recruits, number of coral recruits found on each substrate category; N CCRA spp., number of CCRA species included in each substrate category; Morphological Characteristics, characteristic features for each substrate category. FP: fluorescence photography; VLP, visible light photography.”

**Pg13 ln 245 – I do not understand why two separate Kruskal-Wallis tests were used. It seems like they were testing the same thing to see if there is a difference in substrate/CCRA cover across the tiles. Why isn’t this just one test?**

Thank you for this question and we have omitted the second Kruskal-Wallis test (lines 263-266).

“A Kruskal-Wallis H test followed by a Tukey’s Honest Significant Difference post-hoc test examined if the cover of CCRA substrate categories differed significantly from each other.”

**Results
Pg14 ln 270 – Did any of the sequences match anything on the internal database (collected from natural reef systems in Guam) that was mentioned in the abstract**

Great question and surprisingly only two sequences matched our internal database. We bring this up in lines 290-293.

“Of the 27 putative species, one Corallinales species (Lithophyllaceae sp. 6) and one Peyssonneliales species (*Polystrata* sp. 3) matched the sequence of a CCRA that was previously collected from its natural habitat on Guam’s reefs.”

**Pg17 ln 291 – Why is figure 4 referenced here? Seems like it should be table 1**

Thanks for pointing that out. S1 Table was meant to be referenced here. The reference of figure 4 was deleted (line 285).

“The 84 samples represented 27 distinct species (S1 Table). Of these 27 species, 17 species belonged to the order Corallinales and 10 were members of the order Peyssonneliales.”

**Pg15 Ln 293-298 – Some of this seems like discussion material. Perhaps just retain the first sentence about why you called them Lithophylloideae spp. and save the rest for the discussion**

Great point, thank you. This revision explains why we refer to these species were named to the subfamily level rather than the genus level (lines 314-320).

“Four of the putative species in the Lithophyllaceae belong to the subfamily Lithophylloideae (Fig 2). Lithophylloideae sp. 3 and Lithophylloideae sp. 4 are sister taxa to *Lithophyllum pustulatum* (J.V.Lamouroux) Nägeli and two other *Titanoderma* spp. Lithophylloideae sp. 1 and Lithophylloideae sp. 2 are members of a genus that warrants description. Lithophylloideae sp. 1 was represented by the most sequences in this study. Further phylogenetic analyses are required to resolve the taxonomy of these species and they were provisionally named as distinct species within the subfamily.”

We further explain the discretion to identify these species to the genus level in the discussion (lines 490-500).

“The validity of the genus *Titanoderma* has been debated [86,87] and therefore the species from the recruitment tiles are referred to as Lithophylloideae spp. Lithophylloideae sp. 1 and Lithophylloideae sp. 2 are sister taxa to species named *Titanoderma* sp. and *Lithothrix* sp. in Peña et al. [71] (Fig 2). However, the type species *Titanoderma* *pustulatum*, as *Lithophyllum pustulatum* (J.V.Lamouroux) Foslie *sensu* Peña et al. [71] (Fig 2), was resolved in a different clade. Our results suggest that the description of a new genus is warranted for the clade that contains Lithophylloideae spp. 1–2 and other taxa identified in the literature as *Titanoderma*, but is paraphyletic to the clade that contains *T. pustulatum* [46,71,88]. Further investigations into and a detailed description of Lithophylloideae spp. 1–2 is appropriate given the important ecological role of Lithophylloideae sp. 1 as a recruitment substrate for coral larvae.”

**Pg17 ln 334 – Fig 3 caption – Your methods say you done a maximum likelihood tree, but the figure caption says it is a Bayesian tree. Which is it?**

Thank you, a Maximum likelihood phylogeny was inferred for the 10 Peyssonneliales species. The Bayesian tree statement was deleted.

**Pg17 ln339 – Make this heading bold font**

Thank you, the font was changed to bold.

**Pg18 ln 365 – Please add the results of all statistical tests as a table in supplementary material**

Thank you, we agree. S4 Table was created to show the statistical tests.

**Pg19 ln. 366-67 – Please explain what you mean that the substrate categories fell into three groups based on their percentage cover. I don’t think you are trying to group substrate categories based on percentage cover, are you? You are just trying to determine if there is a difference in percentage cover between species.**

Thank you, the 1^st^ paragraph of this section was rewritten to explain the how the ten substrate categories were placed into either Corallinales or Peyssonneliales (lines 358-366). Since the majority of the Corallinales species could not be visually discerned from one another, we felt it was important to point out that as a whole Corallinales members had the highest cover.

“The 27 CCRA species were grouped into ten substrate categories based on their phylogenetic placement and morphological similarities (Figs 2,3; Table 1). Cover and community composition of all twelve tiles was comparable and all tiles were dominated by CCRA that did not show signs of pigmentation loss or bleaching (Fig 5). Corallinales members constituted the highest percent cover on the tiles (>50%; Fig 4; S4 Table), followed by bare substrate (~25%), and Peyssonneliales constituted the lowest cover (<20%; Fig 4; S4 Table). The tiles contained more Corallinales species than Peyssonneliales species. The different Corallinales species were more difficult to discern morphologically than the Peyssonneliales species.”

**Pg19 ln 371 – This sentence contradicts the one below. You say settlement doesn’t differ from random and then go onto say it does. The wording of the G-test results is confusing.**

Thank you, we agree that the way this statement was written in the first manuscript was contradicting. This manuscript includes a revision of the original statement (lines 384-387).

“Tukey’s post hoc test concluded that the CCRA community composition of all 12 tiles did not differ significantly (Fig 5). A significant difference in the percent cover between CCRA substrate categories was found. Lithophylloideae sp. 1 was the dominant species (41.5 +/- 8.5%) in the CCRA communities that covered the tiles (*p* < 0.0001; S4 Table).”

**Pg19 ln 374-75 – You state that you ran multiple G tests in the results. Were these G-test p-values adjusted for multiple comparisons with something like a Bonferroni correction for multiple comparisons**

Thank you for the suggestion to use the something like a Bonferroni correction. We felt that it was important to assess each variable (substrate category) and the Bonferroni corrections would not provide the statistical detail. We did rewrite this section to clarify why separate G-tests were run.

“The G-test for goodness of fit showed that *A. surculosa* larval settlement was not random and that certain substrate categories were favored for recruitment (*p* < 0.001). The one G-test did not provide detail as to which substrate categories were significantly preferred for settlement. Each substrate category was then individually tested for recruitment preference using the G-test.”

**Discussion
Pg20 ln404 - Perhaps adjust sentence to read “…substrates of A. surculosa in the western Pacific and if there was species specific settlement occurring.”**

Thank you, this sentence was rewritten from western Pacific to Guam (line 418-420).

“Prior to this study, it was unclear which CCRA species served as the preferred recruitment substrates of *A. surculosa* in Guam.”

**Pg21 ln418 – Can you expand on what you think the use of recruitment tiles had on the results of this study. Is there important CCRA species for coral settlement that may have been overlooked? Is it likely that CCRA from certain habitats settled on the tiles? Perhaps cite Kennedy et al. 2017 that looked at the use of different recruitment tiles in relation to natural communities.
Kennedy, E. V., Ordoñez, A., Lewis, B. E., & Diaz-Pulido, G. (2017). Comparison of recruitment tile materials for monitoring coralline algae responses to a changing climate. Marine Ecology Progress Series, 569, 129-144.**

That is a wonderful suggestion. This paragraph was expanded to describe that the shape, material, and microfeatures of the recruitment tiles used in our study may have influenced what type of CCRA community developed. Kennedy et al. (2017) was referenced here to provide how different recruitment material may influence what type of CCRA community may develop (lines 461-470).

“CCRA community composition, on the other hand, might be a reliable indicator for habitat, environmental conditions, depth zone as evidenced by the surprising similarity in CCRA communities on each of the recruitment tiles. The material, shape, and microfeatures of the recruitment tiles could have influenced the type of CCRA community that developed. Kennedy et al. [81] studied the recruitment and calcification of crustose coralline algae on six different experimental tiles (including ceramic, plastic, and glass tiles) on three different habitats (backreef, forereef crest, and reef slope) at two orientations (horizontal and vertical) and found that community composition varied between tile material and orientation.”

**Pg21 ln421 – Was the study on Guam’s seaweed flora based on molecular identifications or morphology? If they were based on morphology alone, is it likely that some of your identified species match with these and further investigation of the type specimens is needed?**

Great question! Another manuscript describing Guam’s CCRA community using molecular tools is being submitted for publication that will compare and discuss the islands previous marine flora identification. We realize that we did not make it clear in the last manuscript about how Guam’s seaweed flora had been previously identified. Lines 439-463 discuss Guam’s published flora and how this study differs.

“The most recent account of CCRA diversity for Guam was summarized in a checklist of Guam’s seaweed flora [80], which lists 24 CCRA species for Guam based on morphological identification. Of these 24 species, 17 belong to the Corallinales, two belong to the Peyssonneliales, four belong to the Hapalidiales, and one belongs to the Sporolithales. Recently, two new species records and four new species of *Ramicrusta* (Peyssonneliales) were reported and described for Guam through molecular-assisted alpha taxonomy [58], increasing Guam’s CCRA species count to 30. All of the 27 species on the recruitment tiles are representatives of the Corallinales and Peyssonneliales, resulting in a more than two-fold increase in the known species number of these orders for Guam [80]. It is unlikely that the 27 CCRA species identified in this study correspond to those reported in Lobban & Tsuda [80], as the previously reported taxa are quite distinctive and differ morphologically from the crusts in this study. Furthermore, an ongoing DNA barcoding study of Guam’s CCRA flora reveals that many of the reported species are likely misidentifications as. It is noteworthy to mention that for each of the 13 non-Lithophylloideae Corallinales species only one specimen was sequenced, which complicated their morphological identification in the photographs of the community analysis. Without DNA barcoding, the number of CCRA taxa recognized on the tiles would have been reduced and a reliable assessment of the CCRA taxa that were favored by coral larvae might have been compromised [40]. These results also suggest that cryptic diversity in CCRA around Guam is rampant.”

**Pg21 ln428 – Change “doubling” to “double”**

Reworded “doubling” to “two-fold increase” (line 446-448).

“All of the 27 species on the recruitment tiles are representatives of the Corallinales and Peyssonneliales, resulting in a more than two-fold increase in the known species number of these orders for Guam [80].”

**Pg22 ln433-34 – This is a good statement about how the diversity on the tiles would have been limited with morphological approaches alone. This could be expanded on and state that the if morphology alone had been used it would have affected the reliability of the coral settlement results**

Thank you. We expanded on this statement

lines 441-443: “Without DNA barcoding, the number of CCRA taxa recognized on the tiles would have been reduced and a reliable assessment of the CCRA taxa that were favored by coral larvae might have been compromised [40]. These results also suggest that cryptic diversity in CCRA around Guam is rampant”

lines 507-512: “The difference between this study and previous studies is the use of DNA barcoding and phylogenetic analyses to identify CCRA, whereas CCRA species identification in previous coral recruitment studies was mainly based on morphology [10,33-35,37-40,90]. Using morphological identifications, *Lithophyllum prototypum* (Foslie) Foslie has been reported to be a favored CCRA for coral larval recruitment throughout the tropics and subtropics [9,32,33,90].”

**Pg22 ln445 – Would you consider your tiles to have high microhabitat diversity? Please expand**

Good question. We hypothesize that the tiles are likely a microhabitat based one what has been observed in turf algal communities. Due to the nature of this study, we did not see variations in tile community composition. We discuss that more thorough studies evaluating the CCRA community compositions and comparing their habitats can happen once Guam’s CCRA flora is well-characterized (lines 470-478).

“Algal turf communities have also been characterized by high species richness at small spatial scales [82,83]. Turf algal communities in Lhaviyani Atoll, Maldives, were found to be species rich and highly variable at microhabitat level (samples were separated by 10 cm) [84]. The CCRA communities of our recruitment tiles contained a similarly high species richness, but community composition was homogeneous between tiles. The latter finding probably results from our replicate study design in which environmental diversity was minimized. A thorough evaluation of CCRA community diversity and their ecological value on Guam’s reefs will only be possible once the island’s CCRA flora is well-characterized.”

**Pg22 ln446-456 – I am not sure what the point that is trying to be made in this paragraph. The first two sentence are a repeat of the results, and I am not sure what the middle two sentences mean (ln 450-454). Either remove this paragraph or reword to clarify the point you are trying to make.**

Thank you for bringing that to our attention. Those sentences within this paragraph were removed to avoid repetition and provide a clearer message (lines 464-478).

**Pg23 ln458 – perhaps rename the heading to “identity of CCRA taxa” as this is more resolving nomenclatural status and names than specie identification. I also think that this could be moved below the settlement section of the discussion.**

Thank you, we renamed the heading to “Identity of CCRA taxa”. We see your point about moving this section, but feel it flows best where it is at. The previous section has a ‘broader’ topic discussing diversity and this section goes more into detail with the nomenclature.

**Pg23 ln467 – You can’t recognize if a species belongs to a genus based on a BLAST search. You need phylogenetic trees to determine these relationships, which you have. Please re-word.**

Thank you for pointing that out. We have reworded the sentence to explain why phylogenetic analyses needed to be performed to determine species relationships (lines 486-488).

**Pg23 ln472 – Is *Titanoderma pustulatum* in the phylogenetic tree (S1 fig)? I don’t see it. Please clarify that Titanoderma pustulatum is the type of the genus and therefore Lithophylloideae sp. 1 & 2 are not *Titanoderma*.**

Great point. It is, if you are accepting *Titanoderma* as a valid genus, it would be *T. pustulatum*. If *Titanoderma* is synonymized under *Lithophyllum*, it is *Lithophyllum pustulatum*. We kept the names that were affiliated with comparison sequences from Peña et al. (2020), in this case the sequence was called *Lithophyllum pustulatum***.** We provide a further explanation of this (lines 490-500). The Lithophylloideae subfamily is in need of phylogenetic attention, but this paper is not about the complexities within the subfamily.

“The validity of the genus *Titanoderma* has been debated [86,87] and therefore the species from the recruitment tiles are referred to as Lithophylloideae spp. Lithophylloideae sp. 1 and Lithophylloideae sp. 2 are sister taxa to species named *Titanoderma* sp. and *Lithothrix* sp. in Peña et al. [71] (Fig 2). However, the type species *Titanoderma* *pustulatum*, as *Lithophyllum pustulatum* (J.V.Lamouroux) Foslie *sensu* Peña et al. [71] (Fig 2), was resolved in a different clade. Our results suggest that the description of a new genus is warranted for the clade that contains Lithophylloideae spp. 1–2 and other taxa identified in the literature as *Titanoderma*, but is paraphyletic to the clade that contains *T. pustulatum* [46,71,88]. Further investigations into and a detailed description of Lithophylloideae spp. 1–2 is appropriate given the important ecological role of Lithophylloideae sp. 1 as a recruitment substrate for coral larvae.”

**Pg24 ln475-488 - I would delete this whole last paragraph as it not relevant. It is important to resolve the identity of the species and apply appropriate names given their ecological importance. However, you did not measure any anatomical characters in this study, and I don’t think you need to get into a discussion about the validity of the genus Titanoderma and anatomical characters used to define it. What you have above this paragraph is sufficient saying more work is needed on the taxonomy.**

Thank you, we agree that this paragraph was not necessary for this manuscript, there for we deleted the paragraph.

**Pg24 ln490 – Please provide citations for the statement in this first sentence. Also, state that this is for other *Acropora* species and not *A. surculosa***

Thank you for bringing that to our attention. We provided citations and clarified that other studies use other *Acropora* species (lines 502-504).

“Despite their ability to recruit on a broad diversity of CCRA species, *Acropora* spp. have been reported to actively favor specific CCRA species as recruitment substrates when present [9,14,32,33].”

**Pg24 ln496 – This would be a good place to bring up that these previous studies were probably based on morphological identifications that could have lumped multiple species under the name *T. prototypum*. Then point out that this is what distinguishes your study.**

Thank you for that suggestion. The revisions clarify that studies have lumped *Lithophyllum prototypum* (*T. prototypum*) in recruitment studies, but this species identification should be taken with reservations. This paragraph points out what distinguishes this study from the previous studies (lines 502-537).

E.g. (line 507): “The difference between this study and previous studies is the use of DNA barcoding and phylogenetic analyses to identify CCRA, whereas CCRA species identification in previous coral recruitment studies was mainly based on morphology [10,33-35,37-40,90].”

**Pg25 ln515 – Do you mean species 1 not species 6?**

Thank you for pointing out this mistake. We corrected 6 to Lithophylloideae sp. 1.

**Pg25 ln519 – I suggest starting this sentence with “For example, …”**

Thank you. We agree that the beginning of the sentence need to be changed, although we opted to use “For example…” (line 547-550).

“This distinct fluorescence signature could result from unique pigments, chemical compounds or microbial communities that enhance successful acroporid recruitment. It has been proposed that successful coral larval recruitment and settlement are a function of the epiphytic microbiome communities and chemical signatures of CCRA [15,34], which may be unique at the species level [39]. **In studies where *L. prototypum* was strongly favored by *Acropora cytherea* larvae for settlement and attachment, the alga displayed a distinct microbial community and characteristic metabolomic fingerprint compared to other CCRA species [39,90].**

**Pg26 ln529 – How does competition explain successful larval recruit of coral on non-favoured CCRA? Please expand**

Thank you for pointing that out. This revision does not discuss competition since our study did not cover that. More studies will have to be conducted to understand the ecology of these CCRA species before understanding their competition for larval settlement.

**Pg27 ln544 – This statement undersells your study. Maybe state that further investigation on post settlement survival could be beneficial for *A. surculosa*.**

Thank you for this suggestion. We took out the portion that undersold our study and included how further studies could be beneficial (lines 564-568).

“Studies in which *L. prototypum* was the favored recruitment substrate [10] found that the post-settlement mortality on other CCRA was higher than on *L. prototypum*. Similar studies could be conducted to assess the long-term survival rate of *A. surculosa* colonies on different substrates, like Lithophylloideae sp. 1.”

**Pg28 ln546-577 – This paragraph is a great conclusion!**

Thank you!

**Reviewer #2**

**Reviewer #2: This manuscript requires major revision. Terms need to be defined. The phylogenetic tree needs to be replaced with the one in supplementary material. The phylogenetic tree requires major revision. All of this explained in more detail on .docx with track changes. This is said to be a multigene tree, but for many taxa not all of the genes are provided. In some cases these genes are from DIFFERENT specimens, whose name may or may not be correctly applied. Only those sequences that can be linked to type sequences of corallines can be believed. Nearly all specimens identified based on morpho-anatomy are suspect. Nowhere is it indicated which sequences are linked to type specimens and which are not. All of this needs to be made clear to the reader, which currently is not done. Many sequences are identified only by a collection number and not by a herbarium accession number. This needs to be made clear to the reader in the Tables.**

Thank you for your suggestions. We agree and replaced fig. 2 with Fig S1. We decided to follow Peña et al. for the alignment due to their knowledge and expertise with coralline algae. The use of this alignment is meant is as a guide for phylogenetic placement for our CCRA species and not a description. The GenBank accession number for each sequence is used in our tables. Table S2 provides the accession numbers for each gene.

**The references here are broadly about red algae and not as relevant to your study. References specifically about CCRAs in the tropics would be better, e.g., Gabrielson et al. 2018 on Porolithon J. Phycol; Hernandez-Kantun et al. 2016 on Lithophyllum J. Phycol., Richards et al. 2017 on Sporolithon J. Phycol.**

Thank you for this suggestion. We agree with you and therefore changed the references to these statements (lines 102-109).

“Investigations using DNA sequencing have revealed that morpho-anatomical identifications of CCRA are often inaccurate [40], because of the specialized techniques required [41-44] and the high degree of cryptic diversity in CCRA [45-51]. Species identification of CCRA can be notoriously challenging due to their simple morphologies, convergent evolution, phenotypic plasticity, and the frequent absence of reproductive structures [43,52]. Despite dispersal limitations, many CCRA species are reported incorrectly to have broad geographical ranges, which are largely based on a morphospecies concept [53].”

**Twist et al. 2020 MEPS would be another good citation here.**

Thank you, we included Twist et al. 2020 in the citations (line 101-102).

**Define this term here when it first is used.**

Thank you for pointing that out. This revision provides more detail about the tiles, CCRA settlement, and coral larval recruitment process. We amended using the term cure.

**This acronym needs to be explained. I did not see a list of abbreviations at the start of the manuscript.**

Thank you pointing that out. We did not use the SECORE aronym, but instead cited the company (lines 130-132).

“Twelve ceramic star-shaped coral recruitment tiles [**56**] were maintained in the flow-through seawater system at the University of Guam Marine Laboratory (UOGML) to recruit CCRA one month prior to the July 2018 *Acropora* spawning event.”

**There is no 'official' barcode marker for red algae. It is one of the barcode markers that is used.**

Thank you for indicating the inaccuracy of that. This revision amended this sentence by explaining that it is a barcode marker that is regularly used for red algae (lines 190-192).

“The mitochondrial cytochrome c oxidase subunit I, COI-5P (roughly 664 bp), is a barcode marker that is regularly used for DNA barcoding of red algae [57] and was used for both the Corallinales and Peyssonneliales.”

**Need citations for these.**

Thank you for indicating that the amplification statement was missing the correct citation. We have inputted citation.

“Amplification of *rbc*L used the primers F57 and rbcLrevNEW [62] following the PCR protocol outlined in Saunders and Moore [62].”

**Incorrect citation.**

Thank you, we fixed the citation.

**Need to define this acronym.**

Thank you for stating that NNI was not defined. Line 222 defines NNI.

“IQ-TREE 2 uses a combination of hill-climbing approaches and stochastic **nearest neighbor interchange (NNI)** operations to obtain higher likelihoods while estimating maximum likelihood phylogenies [68].”

**I find this hard to believe. All of the coralline species? None to family or genus?**

Yes, this was surprising to us as well, but all had >97% sequence similarity to publicly available sequences. Since there are inconsistencies of nomenclature for specimens reported as coralline species, we felt that a phylogenetic approach would be more valid and accurate in each species taxonomic placement for this study.

**This alignment does not consist of 7 genes for the vast majority of the taxa you include. Many are only represented by 3 or fewer genes. This needs to be made clear to the reader. For many of these you are assuming the names in GenBank are correct as the sequences are not from a single specimen. For some of these I know they are not the named taxon that you claim. These need to be checked carefully. I have noted a few on Table S2.**

Thank you for pointing that out. We followed and honored the taxon names and sequences used in Peña et al. since the nomenclature of coralline algae are continuously being refined. We also note that the gene matrix of this alignment is incompletely filled. We are just using this for a guide in the best taxonomic placement available for our CCRA species, we are not describing genera or species. Our reasoning for using the alignment from Peña et al. (2020) is explained in lines 238-245. We concluded to use the names that were used in Peña et al. (2020) since molecular analyses are consistently changing our perspective of the nomenclature for many Corallinales members.

“The concatenated gene matrix of this alignment is incompletely filled, certain taxa are represented by gene sequences from more than one specimen (sometimes from different geographical areas), and some identifications might require nomenclatural updates as CCRA taxonomy is continuously being refined. Yet, we honor the expert opinion of Peña et al. [71] in generating this alignment and the taxonomic identifications that they settled on, particularly because the alignment only serves as a guide for the phylogenetic placement of CCRA specimens from Guam.”

**This sentence needs to be rewritten for clarity. The outgroup already is given in the above sentence.**

Thank you, this sentence was rewritten for clarity and the outgroup was deleted in this sentence to avoid repetition (lines 254-257).

“The sequences used for species divergence of the Peyssonneliales species belonged to the genera *Incendia, Metapeyssonnelia, Peyssonnelia*, *Polystrata*, *Ramicrusta*, *Riquetophycus*, and *Sonderophycus* to help resolve taxonomic identification of the putative species.”

**Needs to be defined in Table title.**

Thank you for asking for clarification on Table 1. We have since made edits to the table and provided a definition in the caption (pg. 9).

**Table 1. Description of the nine substrate categories found on the recruitment tiles.** N Recruits, number of coral recruits found on each substrate category; N CCRA spp., number of CCRA species included in each substrate category; Morphological Characteristics, characteristic features for each substrate category. FP: fluorescence photography; VLP, visible light photography.

**Interesting no Hapalidiales or Sporolithales.**

Yes, that was surprising given the species richness.

**This needs to be Fig. 2. The current Fig. 2 is meaningless to the reader as there is a complete lack of context. Looking at the current Fig. 2, the reader has no idea the clade of Harveylithon species really belong in that genus. The current Fig. 2 can be deleted. What the reader needs to know is all in Fig. S1. That figure needs additional information. No subfamilies are indicated on the tree yet they are presented here. Those names need to be adjacent to their clades on the tree. Also needed is some indication of what sequences are from type specimens or can be linked to type specimens. Maybe place an *before those sequences? The vast majority of the sequences on the tree have only collection numbers and are not accessioned into herbaria. This needs to be made clear in Table S2 that these are personal collection numbers and not herbarium accession numbers. Table S2: Why are some GenBank accession numbers in bold text and others are not? Why is there no locality data for any specimen? And for many entries no collection numbers or herbarium accession numbers are provided. Why aren't generitypes that have been sequenced not in the tree, e.g., Porolithon onkodes, Chamberlainium tumidum?**

Thank you for bringing that to our attention. Fig S1 is now Fig 2. None of the GenBank accession numbers are in bold in this manuscript submission for Table S2. You bring up great points. In this case we followed the same protocols, names, and voucher and accession numbers used in Peña et al. (2020) for the alignment. Lack of locality and collection number is due to certain taxa have sequences from more than one specimen, which we mention in lines 238-245.

“The concatenated gene matrix of this alignment is incompletely filled, certain taxa are represented by gene sequences from more than one specimen (sometimes from different geographical areas), and some identifications might require nomenclatural updates as CCRA taxonomy is continuously being refined.”

**Cite Index Herbariorum for this acronym. And this is confusing. When specimens are cited that are deposited in a herbarium, they are cited by the herbarium acronym followed by the accession number. Is GH part of the accession number? If not, why include? GUAM ######### or GUAM GH#######. GH is the acronym for a completely different herbarium according to Index Herbariorum online.**

Thank you for pointing that out. GH is not part of the accession number. GH is part of the specimen number. Labeling specimens as GH####### is the way this lab identifies and organizes samples. S1 Table has both the voucher number (GH#######) and the accession numbers that correlate with that sample in the collection. The captions for fig 2 and 3 state “GH numbers are the specimens’ voucher numbers in the Guam Herbarium (GUAM).”

**This tree is much more informative than Fig. 2, as sequences from GenBank are included. Again here, type sequences or sequences linked to type sequences need to be indicated on the tree.**

Thank you, the type sequences are indicated in S3 Table.

**Healthy and stressed are relative terms. Please define how you are using these terms when they are first used.**

Thank you for pointing out that is not clear. We deleted the word healthy and replaced it with: “Cover and community composition of all twelve tiles was comparable and all tiles were dominated by CCRA that did not show signs of pigmentation loss or bleaching.” (lines 359--361).

**This is now a third relative term that needs to be defined. What is the difference between unhealthy and stressed?**

Thank you for pointing that out. Instead of using the terms unhealthy and stressed, we said “…a Corallinales spp. that displayed pigmentation loss but was not bleached.” (lines 371-373)

**Can you really extrapolate to the Western Pacific based on 1 study on Guam? I would just write Guam here.**

Replaced Western Pacific to Guam (line 4219-420).

“Prior to this study, it was unclear which CCRA species served as the preferred recruitment substrates of *A. surculosa* in Guam.”

**You have taxa in Corallinales and Peyssonneliales. These orders are not closely related. Delete closely related.**

Thank you for suggesting that. This sentence was rewritten (lines 428-430).

“By sampling thalli for DNA in conjunction with a photographic benthic survey, we were able to describe and quantify the CCRA community composition that was established on coral recruitment tiles.”

**I would state this as a hypothesis and follow up by noting that it could be tested by sequencing the CCRA on the shallow forereef. This would be an interesting study of recruitment over time, as there may be a phenology of spore release.**

Thank you, it would be an interesting study to do over time. We wrote that further studies could indicate if the CCRA species on the recruitment tiles in the flow-through seawater system are a subset of the CCRA flora from the shallow forereef zone in Pago Bay. (lines 431-433)

**Aren't these all DNA sequencing studies. There are lots of different molecules.**

Thank you for stating that. We have rephrased the sentence in line 434.

“The increased use of DNA barcoding in floristic studies has allowed for more accurate and rapid diversity assessments than studies based on morpho-anatomical identifications [40,47,77-79].”

**Twist et al. 2021 could be cited in this paragraph with the caveat that DNA sequencing be expanded to all CCRA.**

Thank you, that was helpful. We do cite Twist et al. in this paragraph (lines 456-458).

“Without DNA barcoding, the number of CCRA taxa recognized on the tiles would have been reduced and a reliable assessment of the CCRA taxa that were favored by coral larvae might have been compromised [40].”

**What are some hypotheses about why this occurred? The rest of the paragraph is about high species diversity across small spatial scales. Does this statement belong here? Maybe move to a different paragraph and present hypotheses and how to test them about why this would occur.**

Thank you for this suggestion. We agree, therefore this topic was moved to the section Ecological significance of CCRA for *Acropora surculosa* recruitment (lines 503-551). In this section we talk about how a previous studies also reported a CCRA species, *L. prototypum (T.prototypum)*, to have a high abundance on recruitment tiles, yet it are not commonly found in the natural reef habitats (line 520-529)

“Despite the dominance of *L. prototypum* on these recruitment tiles, it was not a common CCRA on natural reef systems [9,33].”

Lines 528-538 discuss how the lack of finding/abundance of Lithophylloideae sp. 1 could pose threats to the recovery of reefs.

“Ongoing barcoding efforts of Guam’s CCRA flora have not detected Lithophylloideae sp. 1 on natural reefs yet. Healthy communities of Lithophylloideae sp. 1 would benefit reef recovery following disturbance events that have caused coral die-offs. With growing evidence that many CCRA species have a high level of endemism and restricted distribution ranges [45-51], it is likely that the DNA barcoding of taxa previously identified as *L. prototypum* and *Titanoderma* spp. [9,33,34,90] will reveal a diversity of cryptic species. Based on similarities in ecology and morphology, *L. prototypum* and Lithophylloideae sp. 1 could be closely related taxa of a currently undescribed genus. Further phylogenetic analyses in conjunction with morpho-anatomical observations are required to resolve the diversity and taxonomy of this ecologically important group of CCRA.”

We also added that further studies are needed to investigate the abiotic and biotic factors that contribute to settlement of Lithophylloideae sp. 1 and species that have been referred to as *T. prototypum* (lines 539-551).

“This distinct fluorescence signature could result from unique pigments, chemical compounds or microbial communities that enhance successful acroporid recruitment. It has been proposed that successful coral larval recruitment and settlement are a function of the epiphytic microbiome communities and chemical signatures of CCRA [15,34], which may be unique at the species level [39]. In studies where *L. prototypum* was strongly favored by *Acropora cytherea* larvae for settlement and attachment, the alga displayed a distinct microbial community and characteristic metabolomic fingerprint compared to other CCRA species [39,90].”

**Some background information is needed to understand the studies cited here and more detail needs to be provided. Background information that would be helpful for the entire paper: What is the distribution of A. surculosa? How did you identify the recruits of A. surculosa? Were they sequenced? 'The Pacific' is used, but the Pacific is huge. And aren't you really talking about the tropical and maybe subtropical Pacific. Where were these studies you refer to done? Readers should not have to look up this information when you are making comparisons. In these studies, how were the coral species identified? The algal species? If by morpho-anatomy for the latter, how can we trust the identifications? To be able to compare these studies, readers really need to know the specifics of how these studies were conducted. So I found this a very uncritical comparison that left me with more questions than answers. Also the Plos1 readership is wide and varied, so a lot of care is needed with defining terms and having clear explanations, particularly when studies are being compared.**

Thank you for asking for clarification. We included in the known distribution of *A. surculosa* in the introduction (lines 85-88).

“*A. surculosa* has been reported for islands throughout Micronesia. However, its true distribution is unknown due to taxonomic uncertainty and differences in species concepts for corals from the Indo-Pacific region [25,26].”

We also added in that *A. surculosa* were morphologically identified for collection before the spawning event (lines 138-147-145).

“Prior to the 2018 spawning event, *A. surculosa* colonies were collected on July 1, 2018, from Pago Bay and Tanguisson in Guam. *A. surculosa* is a morphologically distinct coral species on Guam’s reefs. *A. surculosa* colonies were transferred to the holding tanks at UOGML. Colonies from Tanguisson spawned first on July 7, 2018, and colonies from Pago Bay spawned on July 9, 2018. On the nights of the spawning event, released gametes, in the form of egg-sperm bundles, were collected from the water surface of the tank.”

DNA sequencing for coral species for taxonomy and identification has not been successful at this point for *Acropora* taxonomy. There is an ongoing investigation of using DNA sequencing for *Acropora* taxonomy.

We clarified that the previous studies of coral larvae to CCRA use different Acroporid and scleractinian corals in their studies (lines 516-520).

“Recruits of *Acropora tenuis* and *Acropora millepora* on *L. prototypum* also have the highest post-settlement survival rates [9]. Ritson et al. [33] observed *L. prototypum* having a lower benthic cover, yet *Acropora palmata* and *Montastraea faveolata* significantly preferred this species over more abundant CCRA in South Water Cay, Belize.”

Throughout this manuscript, we point out that this study differs from previous studies of coral larvae recruitment preference to CCRA because we used DNA sequencing for identification rather than morphological, which is not reliable. We point this out in the abstract and the ‘Ecological significance of CCRA for *Acropora surculosa* recruitment’ section of the discussion

Lines 16-26: “Most studies on the settlement preference of coral larvae on CCRA use morphological species identifications that are limited by the detection of morphological features, the similarity in morphological characters, the unreliable presence of reproductive structures, phenotypic plasticity, and cryptic diversity. Accurate identification of CCRA species is important for coral reef restoration and management to assess CCRA community composition and to detect CCRA species that are favored as coral recruitment substrates. In this study, DNA sequence analysis was used to identify CCRA species to (1) investigate the species richness and community composition of CCRA on experimental coral recruitment tiles and (2) assess if the coral *Acropora surculosa* preferred any of these CCRA species as recruitment substrates.”

Lines (508-511): “The difference between this study and previous studies is the use of DNA barcoding and phylogenetic analyses to identify CCRA, whereas CCRA species identification in previous coral recruitment studies was mainly based on morphology [10,33-35,37-40,90].”

Line 524: “Since *L. prototypum* identifications in these studies were based on morphology [9,33,34,90], they should be taken with reservation.”

**Only one study is cited at the end of this sentence. And where was this study conducted?**

Thank you, we agree. The sentence was reworded and more citations were added. (lines 511-513)

”Using morphological identifications, *Lithophyllum prototypum* (Foslie) Foslie has been reported to be a favored CCRA for coral larval recruitment throughout the tropics and subtropics [9,32,33,90]. “

We also included descriptions of where these previous studies were conducted throughout the paragraph.

Lines 517-522: “Ritson et al. [33] observed *L. prototypum* having a lower benthic cover, yet *Acropora palmata* and *Montastraea faveolata* significantly preferred this species over more abundant CCRA in South Water Cay, Belize. In an *in-situ* experimental study in Moorea, *L. prototypum* was the dominant of five CCRA species on coral recruitment tiles and was the most favored CCRA species for the recruitment and settlement of *Acropora* and *Pocillopora* larvae [33].”

**Tell the readers where this is in the Pacific.**

Thank you. The localities of the other studies were included.

Lines 517-522: “Ritson et al. [33] observed *L. prototypum* having a lower benthic cover, yet *Acropora palmata* and *Montastraea faveolata* significantly preferred this species over more abundant CCRA in South Water Cay, Belize. In an *in-situ* experimental study in Moorea, *L. prototypum* was the dominant of five CCRA species on coral recruitment tiles and was the most favored CCRA species for the recruitment and settlement of *Acropora* and *Pocillopora* larvae [33].”

**Does T. prototypum occur in Guam? What is its distribution? Did the authors hypothesize why this species was dominant, and what do you mean by dominant? Greatest cover; greatest number of crusts; overgrow other crusts?**

Great question. No, there are no valid reports of *Titanoderma* species have been made for Guam or Micronesia (lines 492-493). This study is not discussing the validity or distribution of *Titanoderma* nor is it aiming to describe a *Titanoderma* species. We felt describing *T. protoypum’s* distribution would take away the message of this study. We briefly bring up the taxonomic complex of *Titanoderma*/*Lithophyllum* (lines 491-502). However, the point of this paper is not to make a claim on the validity of *Titanoderm*a as a genus or not. To prevent confusing the readers, we decided to not go into detail about this nomenclature debate.

“The validity of the genus *Titanoderma* has been debated [86,87] and therefore the species from the recruitment tiles are referred to as Lithophylloideae spp. There are no reports of *Titanoderma* species in Guam or Micronesia. Lithophylloideae sp. 1 and Lithophylloideae sp. 2 are sister taxa to species named *Titanoderma* sp. and *Lithothrix* sp. in Peña et al. [71] (Fig 2). However, the type species *Titanoderma* *pustulatum*, as *Lithophyllum pustulatum* (J.V.Lamouroux) Foslie *sensu* Peña et al. [71] (Fig 2), was resolved in a different clade. Our results suggest that the description of a new genus is warranted for the clade that contains Lithophylloideae spp. 1–2 and other taxa identified in the literature as *Titanoderma*, but is paraphyletic to the clade that contains *T. pustulatum* [46,71,88]. Further investigations into and a detailed description of Lithophylloideae spp. 1–2 is appropriate given the important ecological role of Lithophylloideae sp. 1 as a recruitment substrate for coral larvae.”

Lines 504-539 discusses the ecology of *Titanoderma* spp. from previous studies and how *Titanoderma* spp. had the highest cover (were dominant) in their studies despite being rare in reef environments but are not commonly found in their natural reef habitats. We have similar findings with Lithophylloideae sp. 1 as being dominant on the tiles (percent cover), but despite ongoing efforts it has not been reported for Guam’s reef habitat at this point (lines 528-541).

**vague--give some measurements; larger than the conceptacles of other corallines?**

Thank you, we rewrote this sentence (lines 541-543) as “In visible light, it had a deeper reddish pink pigmentation with noticeably larger conceptacles compared to other CCRA species (Fig 1).”
