# Supplementary material for: Community assessment of crustose calcifying red algae as coral recruitment substrates
Source: PLoS One. 2022 Jul 22;17(7):e0271438. doi: 10.1371/journal.pone.0271438 (PMC9307205; doi:10.1371/journal.pone.0271438)
Supplement: Attachment — Submitted filename: 03_MS.reviewerdocx.docx [file pone.0271438.s011.docx]

Community assessment of crustose calcifying red algae as coral recruitment substrates

Mari E. Deinhart^1*^, Matthew S. Mills^1,2^, Tom Schils^1^

^1^Marine Laboratory, University of Guam, UOG Station, Mangilao, Guam

^2^School of Science, Technology, and Engineering, University of the Sunshine Coast, Sippy Downs, Queensland, Australia

*Corresponding Author

Email: deinhartm@triton.uog.edu

**Keywords**: coral larval settlement, coral reef restoration, CCA, Corallinophycidae, Corallinales, Peyssonneliales, Lithophylloideae, phylogenetics

Abstract

Successful recruitment of invertebrate larvae to reef substrates is essential to the health of tropical coral reef ecosystems and to their capacity to recover from disturbances. Crustose calcifying red algae (CCRA) are a species rich group of seaweeds that have been identified as important recruitment substrates for scleractinian corals. Most studies on the settlement preference of coral larvae on CCRA use morphological species identifications that are limited by the detection of morphological features, the similarity in morphological characters, the unreliable presence of reproductive structures, phenotypic plasticity, and cryptic diversity. Accurate identification of CCRA species is important for coral reef restoration and management to assess CCRA community composition and to detect CCRA species that are favored as coral recruitment substrates. In this study, DNA sequence analysis was used to identify CCRA species to (1) investigate the species richness and community composition of CCRA on experimental coral recruitment tiles and (2) assess if the coral *Acropora surculosa* preferred any of these CCRA species as recruitment substrates. The CCRA community assemblages on the coral recruitment tiles was species-rich, comprising 27 distinct CCRA species of the orders Corallinales and Peyssonneliales. Two closely related CCRA species, Lithophylloideae sp. 1 and Lithophylloideae sp. 2 (Corallinales), were significantly preferred as recruitment substrates over all other CCRA species. Of all CCRA, Lithophylloideae sp. 1 had the highest benthic cover on the recruitment tiles and contained most of the *A. surculosa* recruits. *Titanoderma* species (Lithophylloideae, Corallinales) are typically regarded to be the prime settlement substrates for *Acropora* larvae in the tropical Pacific. The identification of *Titanoderma* taxa in recruitment studies in the Pacific region will be discussed in the context of our ecological and phylogenetic findings. This study reveals a high taxonomic diversity of CCRA communities on a small spatial scale and provides insight into the species-specific ecological functions of CCRA that are often overlooked.

Introduction

Coral reefs are threatened worldwide and are undergoing rapid change because of the increased frequency of coral bleaching events, eutrophication, sedimentation, and pest species outbreaks [1-6]. Apparent declines in live coral cover are generally the first observed warning signs for changes in benthic composition on coral reefs. Many other organisms are intrinsic components of these ecosystems but have received less scientific attention than scleractinian corals. Crustose calcifying red algae (CCRA; representatives of the red algal orders Corallinales, Corallinapetrales, Hapalidiales, Sporolithales, and Peyssonneliales) are a species rich group of macroalgae that are a dominant component of coral reefs. Island groups in the tropical Pacific show significant differences in the abundance of CCRA on their reefs [7], however patterns in CCRA species diversity between these island groups is still largely unknown. CCRA are essential components of healthy reef systems because of their ecological importance as (1) some of the main calcium carbonate producers and carbon sequesters while creating benthic (micro)habitats [8], (2) the preferred settlement substrates for many invertebrate larvae (including scleractinian corals) [9], (3) stabilizers of reef structures [10], and (4) suppressors of nuisance species overgrowth [11-13]. Despite CCRA being among the most dominant benthic organisms on Guam’s tropical reef communities, the diversity and functional ecology of CCRA are still largely unexplored.

CCRA contribute significantly to reef biodiversity [8] and are a dominant benthic group on Guam’s forereef community. When depositing calcium carbonate onto reefs in the form of high-magnesium calcite (Corallinophycidae) or aragonite (Peyssonneliales), CCRA cement and bind reef aggregates together, promote reef growth, provide protection against bioerosion, and enable the settlement of coral and other invertebrate larvae [9-15]. These processes allow CCRA to provide structural complexity and thereby, promote habitat diversity [16].

The coral reefs of Micronesia are known for their high diversity of acroporid corals in the shallow forereef zones [17]. Acroporids are a major group of reef builders due to their high benthic cover and fast growth rates [17,18], thus creating habitat diversity for other reef organisms [19]. Guam’s acroporid corals have undergone extensive mortality in recent years, particularly in the forereef zone, due to repeated bleaching events caused by episodes of elevated seawater temperatures in 2013, 2014, 2016, extreme low tides in 2015, and high predation from *Acanthaster planci* [1-6]. Understanding the factors that drive the recovery of reefs is important as the frequency, severity, and diversity of disturbances impacting coral reefs continue to increase [19]. The recovery of coral reefs depends on the regeneration of coral populations through the successful recruitment of coral larvae [19-22].

The continuing decline in acroporid corals [6] on Guam’s reefs has led to coral restoration efforts that primarily focus on rearing corals in nurseries for outplanting efforts (L. Raymundo, personal communication). Due to its overall resilience to disturbances, *Acropora surculosa* Dana (1846) has been one of the main scleractinian coral species used for restoration efforts in Guam [6]. Corals used for restoration efforts are obtained through fragmentation of source colonies or via sexual reproduction. Sexually produced coral transplants can enhance genetic variability and can generate high numbers of new colonies to be used for large-scale restoration efforts [23,24]. *A. surculosa* has been reported for islands throughout Micronesia. However, its true distribution is unknown due to taxonomic uncertainty and differences in species concepts for corals from the Indo-Pacific region [25,26]. *A. surculosa* is a corymbose acroporid that has been well studied in Guam due to its common occurrence on forereefs, its fast growth rate, and its overall value as a reef builder [27,28]. *A. surculosa* is a hermaphroditic broadcast spawner, with spawning events occurring during the last quarter lunar cycles of July and August [29].

CCRA are well-known to serve as the preferred settlement substrate for coral larvae [9,13,30-34]. Research has largely focused on the specific abiotic and biotic factors that facilitate successful scleractinian coral larval recruitment, settlement, growth, and fecundity [10,14,35]. The University of Guam Marine Laboratory has hosted *s*tudies describing various mechanistic pathways that facilitate *A. surculosa* and *Leptastrea purpurea* larval settlement on CCRA [36-38], but reliable molecular-assisted identifications of the CCRA species that are favored by recruiting *A. surculosa* larvae have not yet been conducted. CCRA species can possess species-specific chemical fingerprints and microbiome communities [39], highlighting the need to correctly identify the CCRA that are favored for larval recruitment in the Micronesian region.

Experimental studies often identify CCRA based on morphological features and often details on the identifications are not reported [40]. Investigations using DNA sequencing have revealed that morpho-anatomical identifications of CCRA are often inaccurate [40], because of the specialized techniques required [41-44] and the high degree of cryptic diversity in CCRA [45-51]. Species identification of CCRA can be notoriously challenging due to their simple morphologies, convergent evolution, phenotypic plasticity, and the frequent absence of reproductive structures [43,52]. Despite dispersal limitations, many CCRA species are reported incorrectly to have broad geographical ranges, which are largely based on a morphospecies concept [53]. Due to these identification challenges, CCRA are often grouped into a single functional group in monitoring and ecological surveys, which does not recognize the species richness of CCRA and the different ecological roles that CCRA species serve in marine environments, including tropical reefs, temperate reefs, and rhodolith beds [54]. To address the challenges of CCRA species identification, DNA sequence analysis has proven to be an effective tool to investigate species diversity in CCRA [48,49,55].

This study used DNA sequencing to investigate the species composition of CCRA communities on coral recruitment tiles in environmental conditions similar to natural reefs with well-developed *Acropora surculose* stands (Pago Bay, Guam). Following the characterization of these CCRA communities, an analysis of recruitment preference by *A. surculose* larvae for CCRA species was conducted. We hypothesized that (1) a diversity assessment using DNA barcoding would reveal more CCRA taxa than a morpho-anatomical diversity assessment and (2) *A. surculosa* larvae prefer to recruit on a select number of CCRA taxa. Based on previous recruitment studies [9,33,34] we hypothesize that *A. surculosa* larvae will favor members of the subfamily Lithophylloideae (Corallinales, Rhodophyta) as recruitment substrates. This study aims to provide new insights on CCRA diversity and community composition and the ecological roles fulfilled by CCRA species.

Materials & Methods

Substrate categories on coral recruitment tiles

Twelve ceramic star-shaped coral recruitment tiles [56] were maintained in the flow-through seawater system at the University of Guam Marine Laboratory (UOGML) to recruit CCRA one month prior to the July 2018 *Acropora* spawning event. The star-shaped tiles allowed for accurate measurements of organism cover on each side of the tiles (excluding the bottom surface). The seawater flowing into the holding tank was drawn from the coral reef at Pago Bay on the east coast of Guam. All twelve coral recruitment tiles were conditioned in the same tank, consequently there was little to no variation in temperature, salinity, turbidity, and water depth between the tiles.

Prior to the 2018 spawning event, *A. surculosa* colonies were collected on July 1, 2018, from Pago Bay and Tanguisson in Guam. *A. surculosa* is a morphologically distinct coral species on Guam’s reefs. *A. surculosa* colonies were transferred to the holding tanks at UOGML. Colonies from Tanguisson spawned first on July 7, 2018, and colonies from Pago Bay spawned on July 9, 2018. On the nights of the spawning event, released gametes, in the form of egg-sperm bundles, were collected from the water surface of the tank. Once gametes were collected, coral colonies were returned to the reef and reattached to minimize damage. After fertilization, embryos were kept in an experimental setting to develop into planula larvae. The larvae were then released into tanks with CCRA-covered tiles for settlement and recruitment.

Following the 2018 *A. surculosa* spawning event, the coral recruitment tiles were maintained in the holding tank for coral larval recruitment. The 12 tiles were taken out of the tank to photograph each of the eleven exposed sides per tile. At the same time, scrapings of live CCRA tissue samples were taken for DNA extraction. Coral recruits were documented while taking photos of the tiles. All CCRA on which coral recruits were detected were sampled for DNA extraction. In addition, 32 samples of CCRA that were not associated with coral recruits were also collected. Samples without associated coral recruits were chosen based on their unique morphology and replicate samples of these morphotypes were taken to address cryptic diversity. Following DNA extraction, tiles were air-dried and deposited into the marine plant collection of the University of Guam Herbarium (GUAM).

For the recruitment preference analysis based on cover data, CCRA taxa were grouped into eight substrate categories that could be discerned visually and included one abiotic category (Table 1). The eight substrate categories included CCRA thalli that were not sequenced but had similar morphological characteristics to CCRA species validated through DNA sequencing analysis. Photographs of each tile and its 11 sides were taken on 13 December 2018. Substrate cover was measured using Adobe Photoshop version CC 2020 software. Color overlays were used to obtain the pixel count for each substrate category present on tile sides. The percent cover for each category was calculated by dividing the total number of pixels of each color (each representing one of the nine categories) by the total pixel count of the tile side (Fig 1). Pixels of coral recruits were ascribed to the substrate category on which they recruited.

**Table 1. Description of the nine substrate categories found on the recruitment tiles.** N Recruits, number of coral recruits found on each substrate category; N CCRA spp., number of CCRA species included in each substrate category; Morphological Characteristics, characteristic features for each substrate category. FP: fluorescence photography; VLP, visible light photography.

| **Substrate Category** | **N Recruits** | | **N CCRA spp.** | **Morphological Characteristics** | |
| --- | --- | --- | --- | --- | --- |
| Corallinales spp. | 3 | 13 | | Bright pink/red in FP. Light pink to light purple in VLP. Conceptacles prevalent but smaller than the conceptacles found on Lithophylloideae sp. 1. |  |
| Lithophylloideae sp. 1 | 45 | 1 | | Salmon red color in VLP. Bright orange in FP. Noticeably large conceptacles. |  |
| Lithophylloideae spp. 2-4 | 5 | 3 | | Magenta in color in VLP. Smaller conceptacles than Lithophylloideae sp. 1. More conceptacles covering the surface area. Deep shade of orange or highlighter pink in FP. |  |
| Peyssonneliales spp. 1,3,6 | 0 | 3 | | Deep red to dark brown color in VLP. Thin, smooth, and firmly attached thallus. |  |
| Peyssonneliales sp. 2 | 0 | 1 | | Thallus is more fleshy, gelatinous, and thick than other Peyssonneliales spp. Bright red in VLP. Bright, light orange color in FP. |  |
| Peyssonneliales spp. 4,7 | 3 | 2 | | Dark red thalli with yellow-olive margins in VLP. Highest variation in color shade and color range compared to other Peyssonneliales categories. |  |
| Peyssonneliales sp. 5 | 0 | 1 | | Lighter shade of red than other Peyssonneliales spp. Thallus appears to be thinner and more firmly attached than other Peyssonneliales spp. |  |
| *Polystrata* spp. 1-3 | 4 | 3 | | Calcified encrusting thallus that is firmly adherent to the substrate. Thallus can be layered. Red in VLP. |  |
| Bare substrate | 0 | 0 | | Bleached or bare substrate with no CCRA or coral growth. |  |

**Fig 1. Example of coral recruitment tile photographs under visible light and actinic light plus the color overlay used for the calculation of percent cover for each substrate category.** Benthic cover was assessed for all 11 exposed sides of the 12 tiles. Substrate category colors are similar to those in Figs 4,5.

DNA barcoding

A total of 92 CCRA specimens were identified using DNA sequencing. For each specimen, a patch of thallus free from epiphytes was swabbed clean with a 10% bleach solution. A Dremel rotary tool, a pair of tweezers, or single-edged razor blades were used to scrape fragments from each specimen for DNA extraction. The Dremel, tweezers, and razor blades were sterilized by soaking them in 10% bleach and heating them over a flame after each tissue removal to avoid contamination. DNA of each CCRA specimen was extracted using QIAGEN DNeasy Blood & Tissue Kit (Qiagen Inc., Valencia, CA) or the GenCatch Blood & Tissue Genomic Mini Prep Kit (Epoch Life Science Inc., Missouri City, TX) following the manufacturer’s bench protocol.

Three genetic markers were amplified via polymerase chain reaction (PCR) for species delimitation and identification. The mitochondrial cytochrome c oxidase subunit I, COI-5P (roughly 664 bp), is a barcode marker that is regularly used for DNA barcoding of red algae [57] and was used for both the Corallinales and Peyssonneliales. The primer combination used to amplify COI-5P was TS_COI_F01_10 (5’- TCGARTCYCGTCTCTCTCG-3’) [58] and the reverse primer, GWSRx [59]. Protocols for COI-5P amplification follow Mills & Schils [58].

Chloroplast photosystem II thylakoid membrane protein D1, *psb*A (roughly 950 bp), was used as a second barcode marker for the Corallinales. P*sb*A is more conserved than COI-5P and is frequently used for CCRA identification because of its high success rate of amplification [60]. The primers used to amplify this gene were psbAF and psbAR2 [61]. Amplification of *psb*A followed the PCR protocol in Mills & Schils [58]. The chloroplast ribulose-1, 5-biphosphate carboxylase large subunit, *rbc*L (roughly 1,350 bp), was amplified for a subset of Corallinales specimens from the coral recruitment tiles. Amplification of *rbc*L used the primers F57 and rbcLrevNEW [62] following the PCR protocol outlined in Saunders and Moore [62].

Species delimitation and phylogenetic analysis

PCR products were sent to Macrogen Inc. (Seoul, Republic of Korea) for DNA sequencing. Once chromatograms were obtained, consensus sequences were assembled using Geneious Pro 11.0.5 computer software (<https://www.geneious.com>; [63]). BLAST searches (Basic local alignment search tool) [64] of the consensus sequences were run to search for close matches in (1) a database of CCRA sequences from Guam and (2) online repositories such as GenBank and the Barcode of Life Database (BOLD) [65]. Gene alignments were created using the MUSCLE plugin [66] in Geneious Pro 11.0.5. An alignment of COI-5P was made for the Peyssonneliales and a concatenated alignment of COI-5P, *psb*A, and *rbc*L for the Corallinales. To assess species richness and to delimit putative species in this study, sequence divergence thresholds were calculated using the Automatic Barcode Gap Discovery (ABGD) [67] for COI-5P (3%), *psb*A (2.5%), and *rbc*L (0.9%) [57].

To resolve the taxonomic identity of CCRA, all sequences from the coral recruitment tiles were selected for phylogenetic analysis. Maximum likelihood (ML) was used to infer phylogenies through IQ-TREE 2 [68]. IQ-TREE 2 uses a combination of hill-climbing approaches and stochastic nearest neighbor interchange (NNI) operations to obtain higher likelihoods while estimating maximum likelihood phylogenies [68]. The concatenated alignment was partitioned by gene and the optimum evolutionary model for each gene was found using ModelFinder [69]. The Ultrafast Bootstrap Approximation was used to achieve unbiased node support values with 1000 replicates [70].

BLAST searches [64] of the Corallinales specimens did not reveal close matches (>97% sequence similarity) with publicly available DNA sequences. Therefore, taxonomic identifications of Corallinales specimens were derived from a phylogeny based on the seven-gene concatenated alignment of Peña et al. [71] (Fig 2), with the addition of the DNA sequences from the recruitment tiles. This alignment of Corallinophycidae (S1 Table; S2 Table; Fig 2) consisted of seven genes (23S rRNA, COI, EF2, LSU rRNA, *psb*A, *rbc*L, and SSU rRNA) and the total length of the seven-gene concatenated alignment was 11,608 bp. The length of the individual gene alignments were: 370 bp for 23S rRNA, 687 bp for COI, 1,622 bp for EF2, 4,716 bp for LSU, 784 bp for *psb*A, 1,386 bp for *rbc*L, and 2,086 bp for SSU. ModelFinder [69] in IQ-TREE 2 [68] found that the best-fit models for each partition were: TVMe+I+G4 (23S rRNA), TN+F+I+G4 (EF2), TIM3+F+I+G4 (LSU), and GTR+F+I+G4 (COI, *psb*A, *rbc*L, and SSU). The concatenated gene matrix of this alignment is incompletely filled, certain taxa are represented by gene sequences from more than one specimen (sometimes from different geographical areas), and some identifications might require nomenclatural updates as CCRA taxonomy is continuously being refined. Yet, we honor the expert opinion of Peña et al. [71] in generating this alignment and the taxonomic identifications that they settled on, particularly because the alignment only serves as a guide for the phylogenetic placement of CCRA specimens from Guam.

BLAST searches of the 10 putative Peyssonneliales species did not resolve their taxonomy either. Therefore, a separate phylogenetic analysis was run based on an alignment of the 10 Peyssonneliales species from the recruitment tiles plus closely related taxa from a BLAST search. The COI-5P sequences of 32 Peyssonneliales specimens from this study were aligned with those of 11 closely related Peyssonneliales taxa (S3 Table) and *Bonnemaisonia asparagoides* (Woodward) C. Agardh as the outgroup. For the closely related taxa, priority was given to sequences of type species within a genus. If sequences of type species were unavailable, sequences of congeners were used. The sequences used for species divergence of the Peyssonneliales species belonged to the genera *Incendia, Metapeyssonnelia, Peyssonnelia*, *Polystrata*, *Ramicrusta*, *Riquetophycus*, and *Sonderophycus* to help resolve taxonomic identification of the putative species. The length of the COI-5P Peyssonneliales alignment was 621 bp. A maximum likelihood phylogeny was generated through IQ-TREE [68]. The best-fit evolutionary model was GTR+F+I+G4 (ModelFinder) [69].

Statistical analysis

Data analysis was carried out in the R environment for statistical computing and visualization (v 3.5.1; R Development Core Team 2020). The percent cover of substrate categories on recruitment tiles was presented as mean ± standard deviation. A Kruskal-Wallis H test followed by a Tukey’s Honest Significant Difference post-hoc test examined if the cover of CCRA substrate categories differed significantly from each other. The Tukey HSD test compared the mean cover of each substrate category to the means of all substrate categories.

The G-test for goodness-of-fit (likelihood ratio or log-likelihood ratio) was used to evaluate whether *Acropora surculosa* larvae (1) were evenly distributed over the recruitment tiles and (2) preferred to recruit on specific substrate categories over others. This G-test was chosen because of the small sample size and the existence of one nominal variable with more than two values (substrate categories). The G-test evaluates if the observed number of coral recruits differs significantly from the expected number of recruits on individual tiles or on each of the substrate categories based on their percent cover. The null hypothesis for tile recruitment preference was that their occurrence was evenly spread over all the tiles. The null hypothesis for substrate recruitment preference was that the number of coral recruits per substrate category was a direct function of the percent cover of each substrate category, *i.e.* random settlement.

Results

Species delimitation

Successful DNA sequences were obtained for 84 of the 89 algal specimens from the coral recruitment tiles, which were all representatives of the red algal class Florideophyceae. Of the 84 successfully sequenced specimens, 52 specimens belonged to the order Corallinales, while 32 specimens were representatives of the order Peyssonneliales. The 84 samples represented 27 distinct species (S1 Table). Of these 27 species, 17 species belonged to the order Corallinales and 10 were members of the order Peyssonneliales. Sequencing all three genetic markers for each putative species did not work reliably, but a minimum of two markers were obtained for 16 Corallinales species. No sequences from GenBank or BOLD matched (>97% sequence similarity) the 27 CCRA species from the coral recruitment tiles. Of the 27 putative species, one Corallinales species (Lithophyllaceae sp. 6) and one Peyssonneliales species (*Polystrata* sp. 3) matched the sequence of a CCRA that was previously collected from its natural habitat on Guam’s reefs.

Five specimens could not be sequenced but were visually identical to specimens that were successfully sequenced. One of these specimens was identified as Lithophylloideae sp. 1, due to its similarity in habit to the 33 successfully sequenced specimens of this species. A second Corallinales specimen was visually identified as Lithophylloideae sp. 2–4. A third Corallinales specimen corresponded to species that could only be identified to family level, Lithophyllaceae. Two other specimens were a visual match to the taxon Peyssonneliales spp. 1,3,6.

Corallinales

Of the 17 Corallinales species, 16 species were identified as members of the family Lithophyllaceae with representatives for each of the four subfamilies: Lithophylloideae, Hydrolithoideae, Chamberlainoideae, and Metagoniolithoideae (Fig 2). One Corallinales species was identified as a representative of the family Neogoniolithoideae (Corallinaceae, Rhodophyta).

**Fig 2. Seven-gene concatenated maximum likelihood phylogeny for the 17 Corallinales species from the recruitment tiles (boldface font) and reference sequences from Peña et al. [69].** GH numbers are the specimens’ voucher numbers in the Guam Herbarium (GUAM). The tree was inferred by maximum likelihood with IQ-TREE [66]. Support values listed next to each node are ultrafast bootstrap approximations based on 1000 replicates. Scale bar represents nucleotide substitutions per site.

Four of the putative species in the Lithophyllaceae belong to the subfamily Lithophylloideae (Fig 2). Lithophylloideae sp. 3 and Lithophylloideae sp. 4 are sister taxa to *Lithophyllum pustulatum* (J.V.Lamouroux) Nägeli and two other *Titanoderma* spp. Lithophylloideae sp. 1 and Lithophylloideae sp. 2 are members of a genus that warrants description. Lithophylloideae sp. 1 was represented by the most sequences in this study. Further phylogenetic analyses are required to resolve the taxonomy of these species and they were provisionally named as distinct species within the subfamily.

Six Corallinales species were confidently assigned to genus level. One species was well-resolved within the genus *Hydrolithon* (Hydrolithoideae, Rhodophyta; Fig 2). Four CCRA species belonged to the subfamily Metagoniolithoideae (Fig 2): *Harveylithon* sp. 1, *Harveylithon* sp. 2, *Harveylithon* sp. 3, and *Porolithon* sp. 1. All four species grouped with high support with their congeners. *Harveylithon* sp. 2 and *Harveylithon* sp. 3 are sister taxa, while *Harveylithon* sp. 1 is sister to a *Harveylithon* sp. from Vanuatu [71] (Fig 2). *Neogoniolithon* sp. 1 was the only species in this study that represented the family Corallinaceae (Fig 2) and was well-resolved within the *Neogoniolithon* clade (Fig 2).

Seven Corallinales species could not be assigned to a subfamily (Fig 2). Lithophyllaceae spp. 1–6 could not be assigned to a recognized genus or subfamily (Fig 2). These six species form a clade within the Lithophyllaceae and sister to the Metagoniolithoideae (Fig 2). Therefore, these taxa were referred to as distinct species within the family Lithophyllaceae, i.e. Lithophyllaceae spp. 1–6. Each of these Lithophyllaceae spp. was represented by just a single sample, with at least 2 successfully sequenced markers. Lithophyllaceae sp. 7 also did not group with any other clade. The nearest clade to Lithophyllaceae sp. 7 was the subfamily Hydrolithoideae and the recently described genus *Parvicellularium* [72] (Fig 2). Lithophyllaceae sp. 7 was a singleton specimen with only a successful *psb*A sequence.

Peyssonneliales

Species delimitation of the 32 COI-5P sequences from Peyssonneliales specimens identified 10 distinct species. Three of these species were representatives of the genus *Polystrata* and seven species belonged to two distinct clades that could not be assigned to a recognized genus within Peyssonneliales (Fig 3). Peyssonneliales spp. 1–7 did not match nor were closely related to any sequenced species through a BLAST search. These seven Peyssonneliales members are not part of the clade with the type species of *Peyssonnelia*, *Peyssonnelia squamaria* Gmelin. Peyssonneliales sp. 1 and Peyssonneliales sp. 2 form a strongly supported clade. Peyssonneliales spp. 3–7 were resolved in another clade, with strong support for the subclade consisting of Peyssonneliales spp. 3–6 (Fig 3). Three *Polystrata* species were identified on the coral recruitment tiles (Fig 3).

**Fig 3. Maximum likelihood phylogeny of the 10 Peyssonneliales species (boldface font) from the recruitment tiles and 10 reference sequences from GenBank inferred from COI-5P sequences.** The tree was inferred by maximum likelihood with IQ-TREE [66]. Support values listed next to each node are ultrafast bootstrap approximations based on 1000 replicates. Scale bar represents nucleotide substitutions per site.

Substrate composition and *Acropora surculosa* settlement preference

The 27 CCRA species were grouped into ten substrate categories based on their phylogenetic placement and morphological similarities (Figs 2,3; Table 1). Cover and community composition of all twelve tiles was comparable and all tiles were dominated by CCRA that did not show signs of pigmentation loss or bleaching (Fig 5). Corallinales members constituted the highest percent cover on the tiles (>50%; Fig 4; S4 Table), followed by bare substrate (~25%), and Peyssonneliales constituted the lowest cover (<20%; Fig 4; S4 Table). The tiles contained more Corallinales species than Peyssonneliales species. The different Corallinales species were more difficult to discern morphologically than the Peyssonneliales species.

During the image analysis for substrate cover measurement, it was noted that four small coral recruits had been overlooked during the CCRA sampling process. The CCRA species to which these corals had recruited (*i.e.*, Corallinales spp. and Lithophylloideae sp. 1) were confidently identified in the digital images and were added to the dataset prior to analysis. All coral recruits were associated with CCRA. One coral recruit was associated with a Corallinales spp. that displayed pigmentation loss but was not bleached.

**Fig 4. Box plot of the percent cover for each substrate category on the 12 coral recruitment tiles**. The abscissa lists substrate categories, the ordinate shows percent cover. Median cover of a substrate category is indicated by a horizontal line in the interior of the box. The box represents the inter-quartile range (IQR) between the upper and lower quartiles. Whiskers indicate the minimum and maximum values beyond the IQR. Letters indicate non-significant differences between island groups (*P*>0.05). Box colors match substrate category colors in Figs 1,5.

**Fig 5. Substrate community composition of the 12 coral recruitment tiles.** The abscissa lists the 12 recruitment tiles, the ordinate shows percent cover. Colors match substrate category colors in Figs 1,4.

Tukey’s post hoc test concluded that the CCRA community composition of all 12 tiles did not differ significantly (Fig 5). A significant difference in the percent cover between CCRA substrate categories was found. Lithophylloideae sp. 1 was the dominant species (41.5 +/- 8.5%) in the CCRA communities that covered the tiles (*p* < 0.0001; S4 Table). The number of coral recruits on each of the twelve tiles did not differ significantly from random recruitment. The G-test for goodness of fit showed that *A. surculosa* larval settlement was not random and that certain substrate categories were favored for recruitment (*p* < 0.001). Each substrate category was then individually tested for recruitment preference using the G-test. A total of 33 samples of Lithophylloideae sp. 1 were successfully sequenced from the coral recruitment tiles, which was consistently the dominant species on the tiles (Fig 4; S4 Table). The habit of Lithophylloideae sp. 1 was distinctly recognizable in the photo analysis and allowed for calculating its percent cover. Of the 60 coral recruits on the tiles, 45 recruited on Lithophylloideae sp. 1 (Table 2). Several of the sequenced Lithophylloideae sp. 1 samples had more than one coral recruit associated with them. The G-test clarified that the higher number of coral recruits on Lithophylloideae sp. 1 was more than just the result of the high percent cover of this species. *A. surculosa* larvae significantly preferred this species as a recruitment substrate (Table 2; *p* < 0.0001; S4 Table)*.*

Lithophylloideae spp. 2–4 was the substrate category with the second most coral recruits (S4 Table). The average surface area of Lithophylloideae spp. 2–4 was 2.9% per tile (Fig 4). Despite its low cover, *A. surculosa* larvae also significantly preferred to recruit on this substrate category (*p* = 0.037; S4 Table). Of the seven remaining substrate categories, three had coral recruits on them: Corallinales spp., Peyssonneliales spp. 4,7 and *Polystrata* spp. 1–3. Corallinales spp. was the category that comprised the highest number of species (13 spp.; Table 1). Many of the species were represented by a single specimen. Corallinales spp. comprised a large percent cover on the recruitment tiles (31.9%; Fig 4) but the number of corals that recruited on this category was significantly lower than expected based on its percent cover (*p* < 0.0001; S4 Table). The remaining two categories with coral larval recruits were not statistically significantly preferred as recruitment substrates: Peyssonneliales spp. 4,7 had four recruits (6.8% cover; *p* = 0.977; S4 Table) and *Polystrata* spp. 1–3 had three recruits (3.8% cover; *p* = 0.650; S4 Table).

Discussion

Successful dispersal and recruitment of invertebrate larvae are crucial to the health and recovery of tropical reef ecosystems following environmental disturbance [73]. Prior to this study, it was unclear which CCRA species served as the preferred recruitment substrates of *A. surculosa* in Guam. This study reports on (1) the diverse CCRA community composition of newly colonized bare substrates in a semi-natural environment and (2) the CCRA species that were significantly favored by *A. surculosa* recruits*.*

CCRA species diversity and community composition

CCRA are a speciose and phylogenetically diverse group that is often treated as a single functional group in ecological and experimental studies. Studies on the taxonomic and ecological composition of CCRA communities are scarce [74] and are typically based on morphological observations [31-33,75,76]. By sampling thalli for DNA in conjunction with a photographic benthic survey, we were able to describe and quantify the CCRA community composition that was established on coral recruitment tiles. Further studies could indicate if the CCRA species on the recruitment tiles in the flow-through seawater system are a subset of the CCRA flora from the shallow forereef zone in Pago Bay.

The increased use of DNA barcoding in floristic studies has allowed for more accurate and rapid diversity assessments than studies based on morpho-anatomical identifications [40,47,77-79]. For example, recently 72 new records of algal species, including members of the Corallinales and Peyssonneliales, were reported for four shallow reef sites in northern Madagascar [79]. Similarly, DNA barcoding documented 122 CCRA species from various sites around New Zealand and identified CCRA diversity hot spots around the island [48]. The most recent account of CCRA diversity for Guam was summarized in a checklist of Guam’s seaweed flora [80], which lists 24 CCRA species for Guam based on morphological identification. Of these 24 species, 17 belong to the Corallinales, two belong to the Peyssonneliales, four belong to the Hapalidiales, and one belongs to the Sporolithales. Recently, two new species records and four new species of *Ramicrusta* (Peyssonneliales) were reported and described for Guam through molecular-assisted alpha taxonomy [58], increasing Guam’s CCRA species count to 30. All of the 27 species on the recruitment tiles are representatives of the Corallinales and Peyssonneliales, resulting in a more than two-fold increase in the known species number of these orders for Guam [80]. It is unlikely that the 27 CCRA species identified in this study correspond to those reported in Lobban & Tsuda [80], as the previously reported taxa are quite distinctive and differ morphologically from the crusts in this study. Furthermore, an ongoing DNA barcoding study of Guam’s CCRA flora reveals that many of the reported species are likely misidentifications. It is noteworthy to mention that for each of the 13 non-Lithophylloideae Corallinales species only one specimen was sequenced, which complicated their morphological identification in the photographs of the community analysis. Without DNA barcoding, the number of CCRA taxa recognized on the tiles would have been reduced and a reliable assessment of the CCRA taxa that were favored by coral larvae might have been compromised [40]. These results also suggest that cryptic diversity in CCRA around Guam is rampant.

The small surface area from which our 92 CCRA samples were collected (106 cm^2^ per tile) further supports the data that CCRA species richness at small spatial scales can be hyper-diverse, even on substrates with little to no microhabitat diversity. CCRA community composition, on the other hand, might be a reliable indicator for habitat, environmental conditions, depth zone as evidenced by the surprising similarity in CCRA communities on each of the recruitment tiles. The material, shape, and microfeatures of the recruitment tiles could have influenced the type of CCRA community that developed. Kennedy et al. [81] studied the recruitment and calcification of crustose coralline algae on six different experimental tiles (including ceramic, plastic, and glass tiles) on three different habitats (backreef, forereef crest, and reef slope) at two orientations (horizontal and vertical) and found that community composition varied between tile material and orientation. Algal turf communities have also been characterized by high species richness at small spatial scales [82,83]. Turf algal communities in Lhaviyani Atoll, Maldives, were found to be species rich and highly variable at microhabitat level (samples were separated by 10 cm) [84]. The CCRA communities of our recruitment tiles contained a similarly high species richness, but community composition was homogeneous between tiles. The latter finding probably results from our replicate study design in which environmental diversity was minimized. A thorough evaluation of CCRA community diversity and their ecological value on Guam’s reefs will only be possible once the island’s CCRA flora is well-characterized.

Identity of CCRA taxa

Species delineation based on DNA barcoding and phylogenetics has become standard practice in phycology, allowing for the rapid identification and detection of (new) species [85]. While this approach has broadened our understanding of algal diversity, it has created challenges to describe and name algal species, including CCRA, since formal descriptions are time-consuming and trail behind the molecular identification of species [44]. Of the 27 species identified, seven Peyssonneliales species and nine Corallinales species could not be reliably assigned to recognized genera. Upon completion of BLAST searches, these 16 species were not closely matched (>97%) with publicly available DNA sequences. Therefore, phylogenies were used to identify species to the lowest taxonomic level possible.

The validity of the genus *Titanoderma* has been debated [86,87] and therefore the species from the recruitment tiles are referred to as Lithophylloideae spp. There are no reports of *Titanoderma* species in Guam or Micronesia. Lithophylloideae sp. 1 and Lithophylloideae sp. 2 are sister taxa to species named *Titanoderma* sp. and *Lithothrix* sp. in Peña et al. [71] (Fig 2). However, the type species *Titanoderma* *pustulatum*, as *Lithophyllum pustulatum* (J.V.Lamouroux) Foslie *sensu* Peña et al. [71] (Fig 2), was resolved in a different clade. Our results suggest that the description of a new genus is warranted for the clade that contains Lithophylloideae spp. 1–2 and other taxa identified in the literature as *Titanoderma*, but is paraphyletic to the clade that contains *T. pustulatum* [46,71,88]. Further investigations into and a detailed description of Lithophylloideae spp. 1–2 is appropriate given the important ecological role of Lithophylloideae sp. 1 as a recruitment substrate for coral larvae.

Ecological significance of CCRA for *Acropora surculosa* recruitment

Despite their ability to recruit on a broad diversity of CCRA species, *Acropora* spp. have been reported to actively favor specific CCRA species as recruitment substrates when present [9,14,32,33]. Prior to this study, it was unknown if and which CCRA species were preferred by *A. surculosa* as recruitment substrates. Here we report that *A. surculosa* larvae actively favor certain CCRA species over others, which supports similar findings for other *Acropora* species [9,32-34]. The difference between this study and previous studies is the use of DNA barcoding and phylogenetic analyses to identify CCRA, whereas CCRA species identification in previous coral recruitment studies was mainly based on morphology [10,33-35,37-40,90]. Using morphological identifications, *Lithophyllum prototypum* (Foslie) Foslie has been reported to be a favored CCRA for coral larval recruitment throughout the tropics and subtropics [9,32,33,90]. On the Great Barrier Reef, settlement of coral recruits on *L. prototypum* has been reported to be 15 times higher than a less preferred species, *Neogoniolithon fosliei* (Heydrich) Setchell & L.R.Mason. Recruits of *Acropora tenuis* and *Acropora millepora* on *L. prototypum* also have the highest post-settlement survival rates [9]. Ritson et al. [33] observed *L. prototypum* having a lower benthic cover, yet *Acropora palmata* and *Montastraea faveolata* significantly preferred this species over more abundant CCRA in South Water Cay, Belize. In an *in-situ* experimental study in Moorea, *L. prototypum* was the dominant of five CCRA species on coral recruitment tiles and was the most favored CCRA species for the recruitment and settlement of *Acropora* and *Pocillopora* larvae [33]. Despite the dominance of *L. prototypum* on these recruitment tiles, it was not a common CCRA on natural reef systems [9,33]. Since *L. prototypum* identifications in these studies were based on morphology [9,33,34,90], they should be taken with reservation. *L. prototypum* and other taxa identified as *Titanoderma* spp. are regarded to be a minor component of Pacific reefs (low benthic cover) and have been found in the mid and outer zones of reef systems [33,91]. Ongoing barcoding efforts of Guam’s CCRA flora have not detected Lithophylloideae sp. 1 on natural reefs yet. Healthy communities of Lithophylloideae sp. 1 would benefit reef recovery following disturbance events that have caused coral die-offs. With growing evidence that many CCRA species have a high level of endemism and restricted distribution ranges [45-51], it is likely that the DNA barcoding of taxa previously identified as *L. prototypum* and *Titanoderma* spp. [9,33,34,90] will reveal a diversity of cryptic species. Based on similarities in ecology and morphology, *L. prototypum* and Lithophylloideae sp. 1 could be closely related taxa of a currently undescribed genus. Further phylogenetic analyses in conjunction with morpho-anatomical observations are required to resolve the diversity and taxonomy of this ecologically important group of CCRA.

Lithophylloideae sp. 1 was morphologically distinct from the other 16 Corallinales species identified on the recruitment tiles. In visible light, it had a deeper reddish pink pigmentation with noticeably larger conceptacles compared to other CCRA species (Fig 1). Using fluorescence photography, Lithophylloideae sp. 1 displayed a fluorescent orange pigmentation that was not observed for the other 16 Corallinales species (Fig 1). This distinct fluorescence signature could result from unique pigments, chemical compounds or microbial communities that enhance successful acroporid recruitment. It has been proposed that successful coral larval recruitment and settlement are a function of the epiphytic microbiome communities and chemical signatures of CCRA [15,34], which may be unique at the species level [39]. In studies where *L. prototypum* was strongly favored by *Acropora cytherea* larvae for settlement and attachment, the alga displayed a distinct microbial community and characteristic metabolomic fingerprint compared to other CCRA species [39,90].

Despite the relatively high species richness of CCRA on recruitment tiles, only five CCRA species that were not Lithophylloideae members were associated with coral recruits. *A. surculosa* larvae were able to recruit onto Peyssonneliales sp. 4 , *Polystrata* sp. 3, *Harveylithon* sp. 3, Lithophyllaceae sp. 4, and one Corallinales sp. Successful larval recruitment on non-favored CCRA species is probably due to random settlement, but the many abiotic and biotic factors that contribute to larval settlement and recruitment are understudied.

Ocean acidification can alter the chemical recognition between Corallinales species and coral larvae, which can lead to reduced coral recruitment [92]. Peyssonneliales species are believed to be less susceptible to ocean acidification than Corallinales species [93]. *A. surculo*sa larvae recruited to two Peyssonneliales species (Peyssonneliales sp. 4 and *Polystrata* sp. 3). A study of coral larval recruitment by *Goniastrea retiformis* in Guam demonstrated that the larvae of this coral can recruit to Peyssonneliales species, but they are not the preferred recruitment substrates [31]. Studies in which *L. prototypum* was the favored recruitment substrate [10] found that the post-settlement mortality on other CCRA was higher than on *L. prototypum*. Similar studies could be conducted to assess the long-term survival rate of *A. surculosa* colonies on different substrates, like Lithophylloideae sp. 1.

This study demonstrates that DNA barcoding is an effective tool for the detailed characterization of CCRA communities, which cannot be accomplished using morphological examinations. Given the high CCRA species richness on the small experimental tiles, it is expected that the floristic diversity of CCRA in the tropical Pacific is severely understudied. Experiments that investigate the ecological functions of CCRA are particularly valuable to assess and evaluate reef health in monitoring and environmental impact studies. Understanding that recruitment of acroporid larvae on CCRA is highly species-specific is important to guide coral reef management and conservation programs. The highly favored Lithophylloideae sp. 1 has yet to be described and detected on natural reef systems. Further research on the diversity, community composition, and ecology of CCRA in tropical reef communities is required to build a holistic view of reef ecosystem functioning.

Acknowledgements

We are grateful to Dr. Laurie Raymundo and the Raymundo Coral Lab for the use of their coral recruitment tiles. The manuscript benefitted from conversations and assistance regarding R scripting and data analysis by Andrew McInnis. A special thanks to Dr. Alex Kerr and Dr. Heroen Verbruggen for their invaluable time in extending their knowledge. MD, MM, and TS are indebted to the University of Guam for supporting studies that document and conserve the natural resources of Guam and the greater Micronesian region.

Funding

This research is based upon work supported by the National Aeronautics and Space Administration (NASA; nasa.gov) and the National Science Foundation (NSF; nsf.gov) under grant numbers 80NSSC17M0052 and OIA-1946352 awarded to TS and managed through the Guam EPSCoR offices of NASA and NSF. Any opinions, findings, and conclusions or recommendations expressed in this manuscript are those of the authors and do not necessarily reflect the views of NASA, NSF or any of their subagencies. The funders had no role in study design, data collection and analysis, decision to publish, or preparation of the manuscript.

References

1. Chesher RH. Destruction of Pacific corals by the sea start *Acanthaster planci*. Science. 1969; 165:280–283. doi: 10.1126/science.165.3890.280.
2. Colgan MW. Coral reef recovery on Guam (Micronesia) after catastrophic predation by *Acanthaster Planci*. Ecology. 1987; 68:1592–1605. doi: 10.2307/1939851.
3. Paulay G. Marine biodiversity of Guam and the Marianas: overview. Micronesica. 2003; 36:3–25.
4. Raymundo LJ, Burdick D, Lapacek VA, Miller R, Borwn V. Anomalous temperatures and extreme tides: Guam staghorn *Acropora* succumb to a double threat. Mar Ecol Prog Ser. 2017; doi: 10.3354/meps12005.
5. Maynard J, Johnson SM, Burdick DR, Jarrett A, Gault J, Idechong J, et al. Coral reef resilience to climate change in Guam in 2016. NOAA Coral Reef Conservation Program. NOAA Technical Memorandum CRCP 29, 51 pp. 2017; doi: 10.7289/V5/TM-CRCP-29.
6. Raymundo LJ, Burdick D, Hoot WC, Miller RM, Brown V, Reynolds T, et al. Successive bleaching events cause mass coral mortality in Guam, Micronesia. Coral Reefs. 2019; 38:677–700. doi: 10.1007/s.
7. Schils T, Vroom PS, Tribollet AD. Geographical partitioning of marine macrophyte assemblages in the tropical Pacific: a result of local and regional diversity processes. J Biogeogr. 2013; 40:1266–1277. doi: 10.1111/jbi.12083.
8. Littler MM, Littler DS. Models of tropical reef biogenesis: the contribution of algae. Prog. Phyco. 1984; 3:323-364.
9. Harrington L, Fabricius K, De’ath G, Negri A. Recognition and selection of settlement substrata determine post–settlement survival in corals. Ecology. 2004; 85(12):3428–3437. doi: http://dx.doi.org/10.1890/04-0298.
10. Adey WH. Review—Coral reefs: algal structured and mediated ecosystems in shallow, turbulent, alkaline waters. J. Phycol. 1998; 34:393–406.
11. Keats DW, Wilton P, Maneveldt G (1994) Ecological significance of deep-layer sloughing in the eulittoral zone coralline alga, *Spongites yendoi* (Foslie) Chamberlain (Corallinaceae, Rhodophyta) in South Africa. J Exp Mar Biol Ecol 175:145–154.
12. Keats DW, Knight MA, Pueschel CM (1997) Antifouling effects of epithallial shedding in three crustose coralline algae (Rhodophyta, Coralinales) on a coral reef. J Exp Mar Biol Ecol.213:281–293
13. Gómez-Lemos LA, Diaz-Pulido G. Crustose coralline algae and associated microbial biofilms deter seaweed settlement on coral reefs. Coral Reefs. 2017; 36:453–462. doi: 10.1007/s00338-017-1549-x.
14. Heyward AJ, Negri AP. Natural inducers for coral larval metamorphosis. Coral Reefs. 1999; 18:273–279. doi: 10.1007/s003380050193.
15. Gómez-Lemos LA, Doropoulos C, Bayraktarov E, Diaz-Pulido G. Coralline algal metabolites induce settlement and mediate the inductive effect of epiphytic microbes on coral larvae. Sci. Rep. 2018; 8:1–11. doi: 10.1038/s41598-018-35206-9.
16. Asnaghi V, Thrush SF, Hewitt JE, Mangialajo L, Cattaneo-Vietti R, Chiantore M. Colonisation processes and the role of coralline algae in rocky shore community dynamics. J. Sea Res. 2015; 95:132–138. doi: 10.1016/j.seares.2014.07.012.
17. Raymundo LJ, Burdick D, Lapacek VA, Miller R, Borwn V. Anomalous temperatures and extreme tides: Guam staghorn Acropora succumb to a double threat. Mar Ecol Prog Ser. 2017; doi: 10.3354/meps12005.
18. Bonin MC. Specializing on vulnerable habitat: Acropora selectivity among damselfish recruits and the risk of bleaching-induced habitat loss. Coral Reefs. 2012; 31: 287–297. doi: 10.1007/s00338-011-0843-2.
19. Bellwood DR, Hughes TP, Folke C, Nystrom M. Confronting the coral reef crisis. Nature. 2004; 429: 827. doi: 10.1038/nature02691.
20. Richmond RH. Reproduction and recruitment in corals: Critical links in the persistence of reefs. 175-197 in C. Birkeland. Life and death of coral reefs. Chapman & Hall, New York. 1997; doi: 10.1007/978-1-4615-5995-5_8.
21. Harrison P. (2011). Sexual Reproduction of Scleractinian Corals. Dubinsky Z., Stambler N. (eds) Coral Reefs: An Ecosystem in Transition. Springer, Dordrecht 59–85. doi: 10.1007/978-94-007-0114-4_6.
22. Hughes TP, Kerry JT, Álvarez-Noriega M, Álvarez-Romero JG, Anderson KD, Baird AH, et al. Global warming and recurrent mass bleaching of corals. Nature. 2017; 543:373. doi: 10.1038/nature21707.
23. Heyward AJ, Smith LD, Rees M, Field SN. Enhancement of coral recruitment by in situ mass culture of coral larvae. Mar Eco Prog Ser. 2002; 230:113–118. doi: 10.3354/meps230113.
24. Doropoulos C, Elzinga J, ter Hofstede R, van Koningsveld M, Babcock RC. Optimizing industrial-scale coral reef restoration: comparing harvesting wild coral spawn slicks and transplanting gravid adult colonies. Resto. Ecol. 2019; 27(4):758–767. doi: 10.1111/rec.12918.
25. Randall RH. An annotated checklist of hydrozoan and scleractinian corals collected from Guam and other Mariana Islands. Micronesica. 2003; 35–36:121–137.
26. Hoeksema BW, Cairns S. World List of Scleractinia. *Acropora hyacinthus* (Dana, 1846). World Register of Marine Species; 2021 [cited 2021 Oct 31]. Available from: http://www.marinespecies.org/aphia.php?p=taxdetails&id=207044 on 2021-10-31.
27. Paulay G. Patterns and consequences of coral bleaching in Micronesia (Majuro and Guam) in 1992-1994. Micronesica. 1999; 31(2):109–124.
28. Baker AC, Glynn PW, Riegl B. Climate change and coral reef bleaching: An ecological assessment of long-term impacts, recovery trends and future outlook. Estuar. 2008; 80(4):435–471. doi: 10.1016/j.ecss.2008.09.003.
29. Richmond R, Hunter RH. Reproduction and recruitment of corals: comparisons among the Caribbean, the Tropical Pacific, and the Red Sea. Mar Ecol Prog Ser. 1990; 60:185–203 doi: 10.1007/978-1-4615-5995-5_8.
30. Morse DE, Hooker N, Morse ANC, Jensen R.J Control of larval metamorphosis and recruitment in sympatric agariciid corals. Exp Mar Biol Ecol. 1988; 116:193–217. doi: 10.1016/0022-0981(88)90027-5.
31. Golbuu Y, Richmond RH. Substratum preferences in planula larvae of two species of scleractinian corals, Goniastrea retiformis and Stylaraea punctata. Mar Biol. 2007; doi: 10.1007/s00227-007-0717-x.
32. Price N. Habitat selection, facilitation, and biotic settlement cues affect distribution and performance of coral recruits in French Polynesia. Oecologia. 2010; 163:747–758. doi: 10.1007/s00442-010-1578-4.
33. Ritson-Williams R, Arnold SN, Paul VJ, Steneck RS. Larval settlement preferences of *Acropora palmata* and *Montastraea faveolata* in response to diverse red algae. Coral Reefs. 2014; 33: 59–66. doi: 10.1007/s00338-013-1113-2.
34. Whitman TN, Negri AP, Bourne DG, Randall CJ. Settlement of larvae from four families of corals in response to crustose coralline alga and its biochemical morphogens. Sci. Rep. 2020; 10:16397. doi: 10.1038/s41598-020-73103-2.
35. Ricardo GF, Harper CE, Negri AP, Luter HM, Wahab MAA, Jones RJ. Impacts of water quality on *Acropora* coral settlement: The relative importance of substrate quality and light. Sci Total Environ. 2021; 777: 146079. doi: 10.1016/j.scitotenv.2021.146079.
36. Tebben J, Motti C, Siboni N, Tapiolas D, Negri AP, Schupp P, et al. Chemical mediation of coral larval settlement by crustose coralline algae. Sci. Rep. 2015; 5:10803. doi: 10.1038/srep10803.
37. Moeller M, Niezter S, Schupp PJ. Neuroactive compounds induce larval settlement in the scleractinian coral Leptastrea purpurea. Sci. Rep. 2019; 9: 2291. doi: 10.1038/s41598-019-38794-2.
38. Petersen LE, Moeller M, Versluis D, Niezter S, Kellermann MY, Schupp PJ. Mono- and multispecies biofilms from a crustose coralline alga induce settlement in the scleractinian coral Leptastrea purpurea. Coral Reefs. 2021; 40: 381–394. doi: 10.1007/s00338-021-02062-5.
39. Jorissen H, Galand PE, Bonnard I, Meiling S, Raviglione D, Meistertzheim AL, et al. Coral larval settlement preferences linked to crustose coralline algae with distinct chemical and microbial signatures. Sci. Rep. 2021; 11:14610. doi: 10.1038/s41598-021-94096-6. PMID: 34272460.
40. Twist BA, Cornwall CE, McCoy SJ, Gabrielson PW, Martone PT, Nelson WA. The need to employ reliable and reproducible species identifications in coralline algal research. Mar. Ecol. Prog. Ser. 2020; 654:225–231
41. Abbott I. Vegetative and reproductive morphology in Reticulocaulis gen. nov. and Naccaria hawaiiana sp. nov. (Rhodophyta, Naccariaceae). J Phycol. 1985; 21: 554-561.
42. Woelkerling WJ, Irvine LM, Harvey AS. Growth-forms in non-geniculate coralline red algae (Corallinales, Rhodophyta). Aust. Syst. Bot. 1993; 6:277–293.
43. Harvey AS, Woelkerling WJ. A guide to nongeniculate coralline red algal (Corallinales, Rhodophyta) rhodolith identification. Cienc Mar. 2007; 33(4): 411–426.
44. De Clerck O, Guiry MD, Leliaert F, Samyn Y, Verbruggen H. Algal taxonomy: a road to nowhere? J. Phycol. 2013; 49(2): 215–225. doi: https://doi.org/10.1111/jpy.12020. PMID: 27008509.
45. Richards JL, Gabrielson PW, Fredericq S. New insights into the genus *Lithophyllum* (Lithophylloideae, Corallinaceae, Corallinales) from deepwater rhodolith beds offshore the NW Gulf of Mexico. Phytotaxa. 2014; 190(1): 162–175. doi: http://dx.doi.org/10.11646/phytotaxa.190.1.11 .
46. Richards JL, Gabrielson PW, Hughey PW, Freshwater DW. A re-evaluation of subtidal Lithophyllum species (Corallinales, Rhodophyta) from North Carolina, USA, and the proposal of L. searlesii sp. nov. Phycologia. 2018; 57: 318–330. doi: https://doi.org/10.2216/17-110.1
47. Twist BA, Neill KF, Bilewitch J, Jeong SY, Sutherland JE, Nelson WA. High diversity of coralline algae in New Zealand revealed: Knowledge gaps and implications for future research. PLoS ONE. 2019; 14(12): e0225645. doi: 10.1371/journal.pone.0225645.
48. Sherwood AR, Paiano MO, Spalding HL, Kosaki RK. Biodiversity of Hawaiian Peyssonneliales (Rhodophyta): Sonderophycus copusii sp. nov., a new species from the Northwestern Hawaiian Islands. Algae. 2020; 35(2):145–155. doi: 10.4490/algae.2020.35.5.20.
49. Hernández-Kantún JJ, Riosmena-Rodriguez R, Adey WH, Rindi F. Analysis of the cox2-3 spacer region for population diversity and 70 taxonomic implications in rhodolith-forming species (Rhodophyta: Corallinales). Phytotaxa. 2014; 190(1):331–354. doi: 10.11646/phytotaxa.190.1.20.
50. Hernandez-Kantun JJ, Gabrielson P, Hughey JR, Pezzolesi L, Rindi F, Robinson NM et al. Reassessment of branched *Lithophyllum* spp.(Corallinales, Rhodophyta) in the Caribbean Sea with global implications. Phycologia. 2016; 55(6), 619-639. sepecimens.
51. Gabrielson PW, Hughey JR, Diaz-Pulido G. Genomics reveals abundant speciation in the coral reef building alga Porolithon onkodes (Corallinales, Rhodophyta). J Phycol. 2018; 54(4):429-434.
52. Campbell SJ, Woelkerling WJ. Are *Titanoderma* and *Lithophyllum* (Corallinaceae, Rhodophyta) distinct genera? Phycologia. 1990; 29:114-125. doi:10.2216/i0031-8884-29-1-114.1.
53. Maneveldt, G. W., Gabrielson, P. W., & Kangwe, J. (2017). Sporolithon indopacificum sp. nov.(Sporolithales, Rhodophyta) from tropical western Indian and western Pacific oceans: First report, confirmed by DNA sequence data, of a widely distributed species of Sporolithon. Phytotaxa, 326(2), 115-128.
54. Ford HV, Gove JM, Davies AJ, Graham NAJ, Healey JR, Conklin EJ, et al. Spatial scaling properties of coral reef benthic communities. Ecography. 2020; 44:188-198. doi: 10.1111/ecog.05331.
55. Saunders GW. Applying DNA barcoding to red macroalgae: A preliminary appraisal holds promise for future applications. Philos Trans R Soc Lond B Biol Sci. 2005; 360(1462):1879–1888. doi: 10.1098%2Frstb.2005.1719. PMID: 16214745.
56. SECORE International. (2020). Engineering Restoration, Coral Rearing In-Situ Basins- CRIBS. Available: http://www.secore.org/site/our-work/detail/engineering-restoration.60.html [Accessed April 5, 2021].
57. Torrano-Silva BN, Vieira BR, Riosmena-Rodríguez R, Oliveira MC. Guidelines for DNA barcoding of coralline algae, focusing on Lithophylloideae (Corallinales) from Brazil. Bot Mar. 2018; 61:127–140. doi: 10.1515/BOT-2017-0040.
58. Mills MS, Schils T. The habitat-modifying red algal *Ramicrusta* on Pacific Reefs: a new generic record for the tropical northwestern Pacific and the description of four new species from Guam. PLoS ONE. 2021; 16(11): e0259336. doi: 10.1371/journal.pone.0259336
59. Saunders GW, McDevit DC.Methods for DNA barcoding photosynthetic protists emphasizing the macroalgae and diatoms. Methods Mol. Biol 2012; 858:207-22. doi: 10.1007/978-1-61779-591-6_10.
60. Broom JE, Hart DR, Farr TJ, Nelson WA, Neill KF, Harvey AS, et al. Utility of psbA and nSSU for phylogenetic reconstruction in the Corallinales based on New Zealand taxa. Mol. Phylogenet. Evol. 2008; 46(3), 958-973. doi: 10.1016/j.ympev.2007.12.016.
61. Yoon HS, Hackett JD, Bhattacharya D. A single origin of the peridinin- and fucoxanthin-containing plastids in dinoflagellates through tertiary endosymbiosis. Proc Natl Acad Sci U S A. 2002; 99 (18):11724–11729. doi: 10.1073/pnas.172234799. PMID: 12172008.
62. Saunders GW, Moore TE. Refinements for the amplification and sequencing of red algal DNA barcode and RedToL phylogenetic markers: a summary of current primers, profiles and strategies. Algae. 2013; 2(1):31–43. doi: 10.4490/algae.2013.28.1.031.
63. Kearse M, Moir R, Wilson A, Stones-Havas S, Cheung M, Sturrock S, et al. Geneious Basic: an integrated and extendable desktop software platform for the organization and analysis of sequence data. Bioinformatics. 2012; 28(12):1647–9. doi: 10.1093/bioinformatics/bts199. PMID: 22543367.
64. Altschul SF, Gish W, Miller W, Myers EW, Lipman DJ. Basic local alignment search tool. J. Mol. Biol. 1990; 215(3):403-410. doi: 10.1016/S0022-2836(05)80360-2. PMID:2231712
65. Ratnasingham S, Hebert PDN. BOLD: The barcode of life data system (http://www.barcodinglife.org). Mol Ecol Note. 2007; 7(3):355-364. doi: 10.1111/j.1471-8286.2007.01678.x. PMID: 18784790
66. Edgar RC. MUSCLE: multiple sequence alignment with high accuracy and high throughput. Nucleic Acids Re. 2004; 325(5)1792–1797. doi: 10.1093/nar/gkh340. PMID: 15034147
67. Puillandre N, Lambert A, Brouillet S, Achaz G. ABGD, Automatic Barcode Gap Discovery for primary species delimitation. 2012; 21(8):1864-77. doi: 10.1111/j.1365-294X.2011.05239.x. PMID: 21883587.
68. Minh BQ, Schmidt HA, Chernomor O, Schrempf D, Woodhams MD, von Haeseler A, Lanfear R. IQ-TREE 2: New models and efficient methods for phylogenetic inference in the genomic era. Mol. Biol. Evol. 2020, 37(5):1530-1534. doi: 10.1093/molbev/msaa015. PMID: 32556291
69. Kalyaanamoorthy S, Minh BQ, Wong TKF, von Haeseler A, Jermiin LS. ModelFinder: fast model selection for accurate phylogenetic estimates. Nat Methods. 2017; 14(6):587-589.
70. Hoang DT, Chernomor O, von Haeseler A, Minh BQ, Vinh LS. FBoot2: Improving the Ultrafast Bootstrap Approximation. Mol Biol Evol. 2018; 35(2):518-522. doi: 10.1093/molbev/msx281. PMID: 29077904.
71. Peña V, Vieira C, Braga JC, Aguirre J, Rösler A, Baele G, et al. Radiation of the coralline red algae (Corallinophycidae, Rhodophyta) crown group as inferred from a multilocus time-calibrated phylogeny. Mol Phylogenet Evol. 2020; 150:106845. doi: 10.1016/j.ympev.2020.106845. PMID: 32360706.
72. Caragnano A, Foetisch A, Maneveldt GW, Millet L, Liu LC, Lin SM, et al. Revision of Corallinaceae (Corallinales, Rhodophyta): recognizing Dawsoniolithon gen. nov., Parvicellularium gen. nov. and Chamberlainoideae subfam. nov. containing Chamberlainium gen. nov. and Pneophyllum. J Phycol. 2018; 54(3):391-409. doi: 10.1111/jpy.12644. PMID: 29574890.
73. Hughes TP, Tanner JE. Recruitment failure, life histories, and long-term decline of Caribbean corals. Ecology. 2000; 81(8):2250–2263.
74. Hind KR, Starko S, Burt JM, Lemay MA, Salomon AK, Martone PT. Trophic control of cryptic coralline algal diversity. Proc Natl Acad Sci U S A. 2019; 116(30):15080–15085.
75. Ballesteros E. The deep-water Peyssonnelia Beds from Balearic Islands (Western Mediterranean). Marine Ecology. 1994; 15:233–253. doi: 10.1111/J.1439-0485.1994.TB00055.X.
76. Rindi F, Braga JC, Martin S, Peña V,Le Gall L, Caragnano A, et al. Coralline algae in a changing Mediterranean Sea: how can we predict their future, if we do not know their present? Front. Mar. Sci 2019; 6:723. doi: 10.3389/fmars.2019.00723.
77. Melbourne LA, Hernández-Kantún JJ, Russell S, Brodie J. There is more to maerl than meets the eye: DNA barcoding reveals a new species in Britain, Lithothamnion erinaceum sp. nov. (Hapalidiales, Rhodophyta). Eur J Phycol. 2017; 52:166–178.
78. Kogame K, Uwai S, Anderson RJ, Choi HG, Bolton JJ. DNA barcoding of South African geniculate coralline red algae (Corallinales, Rhodophyta). South African J Bot. 2017; 108:337–341. doi: doi.org/10.1016/j.sajb
79. Vieira C, De Ramon N’Yeurt A, Rasoamanendrika FA, D’Hondt S, Thi Tran LA, Van den Spiegel D, et al. Marine macroalgal biodiversity of northern Madagascar: morpho-genetic systematics and implications of anthropic impacts of conservation. Biodivers Conserv. 2021; doi: 10.1007/s10531-021-02156-0.
80. Lobban CS, Tsuda RT. Revised checklist of benthic marine macroalgae and seagrasses of Guam and Micronesia. Micronesica. 2003; 35(36):5499.
81. Kennedy, E. V., Ordoñez, A., Lewis, B. E., & Diaz-Pulido, G. Comparison of recruitment tile materials for monitoring coralline algae responses to a changing climate. Mar. Ecol. Prog. Ser. 2017; 569:129–144.
82. Connell SD, Foster MS, Airoldi L. What are algal turfs? Towards a better description of turfs. Mar Ecol Prog Ser. 2014; 495:299–307. doi: 10.3354/meps10513.
83. Ober GT, Diaz-Pulido G, Thronber C. Ocean acidification influences the biomass and diversity of reef associated turf algal communities. Mar Biol. 2016; doi: 10.1007/s00227-016-2978-8..
84. Harris JL, Lewis LS, Smith JE. Quantifying scales of spatial variability in algal turf assemblages on coral reefs. Mar Ecol Prog Ser. 2015; 532:41–57. doi: 10.3354/meps11344.
85. Leliaert F. Advancing the science of algal taxonomy. J Phycol. 2021; 57(2):412–415. doi: 10.1111/jpy.13134.
86. Campbell SJ, Woelkerling WJ. Are *Titanoderma* and *Lithophyllum* (Corallinaceae, Rhodophyta) distinct genera? Phycologia. 1990; 29:114-125. doi:10.2216/i0031-8884-29-1-114.1.
87. Chamberlain Y, Irvine LM, Walker R. A redescription of *Lithophyllum orbiculatum* (Rhodophyta, Corallinales) in the British Isles and a reassessment of generic delimitation in the Lithophylloideae. J Phycol. 1991; 26:149–167.
88. Bittner L, Payri CE, Maneveldt GW, Couloux A, Cruaud C, de Reviers B, et al. Evolutionary history of the Corallinales (Corallinophycidae, Rhodophyta) inferred from nuclear, plastid, and mitochondrial genomes. Mol. Phylogenet. 2011; 61 697–713. doi: 10.1016/j.ympev.2008.06.018. PMID: 18652904.
89. Arnold SN, Steneck RS, Mumby PJ. Running the gauntlet: inhibitory effects of algal turfs on the processes of coral recruitment. Mar Ecol Prog Ser. 2010; 414:91–105. doi: 10.3354/meps08724.
90. Dean AJ, Steneck RS, Tager D, Pandolfi JM. Distribution, abundance and diversity of crustose coralline algae on the Great Barrier Reef. Coral Reefs. 2014; 34(4):581-59. doi: 10.1007/s00338-015-1263-5.
91. Siboni N, Abrego D, Puill-Stephan E, King WL, Bourne DG, Raina JB, et al. Crustose coralline algae that promote coral larval settlement harbor distinct surface bacterial communities. Coral Reefs. 2020; doi: 10.1007/s00338-020-01997-5.
92. Doropoulos C, Diaz-Pulido G. High CO_2_ reduces the settlement of a spawning coral on three common species of coralline algae. Mar Ecol Prog Ser. 2013; 475: 93–99. doi: 10.3354/meps10096.
93. Nash MC, Russell BD, Dixon KR, Liu M, Xu H. Discovery of the mineral brucite (magnesium hydroxide) in the tropical calcifying alga Polystrata dura (Peyssonneliales, Rhodophyta). J Phycol., 2015; 51: 403-407. doi: 10.1111/jpy.12299.

Supporting information

**S1 Table.** GenBank accession numbers for COI-5P, *psb*A, and *rbc*L sequences of specimens from the coral recruitment tiles, with indication of the order, taxon name, and herbarium accession number.

**S2 Table.** Taxon list from the Peña et al. [69] sequence alignment utilized to phylogenetically place each Corallinales species identified in this study. The GenBank accession numbers is provided for each gene.

**S3 Table.** GenBank accession numbers or BOLD Systems identification numbers (*) of COI-5P sequences of Peyssonneliales taxa used in Fig 3. Bold taxa names are generitypes.

**S4 Table. G-test for goodness of fit of recruitment preference to substrate categories.** Only substrate categories with coral recruits are listed. The first column lists the substrate categories with associated coral recruits. The second column shows the average precent cover of a substrate category on all tiles. The third column represents the total number of coral recruits associated with a substrate category. The last column shows the probability value of larval recruitment preference.
