# Supplementary material for: Community assessment of crustose calcifying red algae as coral recruitment substrates
Source: PLoS One. 2022 Jul 22;17(7):e0271438. doi: 10.1371/journal.pone.0271438 (PMC9307205; doi:10.1371/journal.pone.0271438)
Supplement: Attachment — Submitted filename: Response to Reviewers.docx [file pone.0271438.s012.docx]

20 June 2022

Dear PLoS ONE Editors,

We thank the editor and the two reviewers for taking the time to provide generous comments and feedback on the manuscript, PONE-D-21-27555, for a second time. Below is our response to each point raised by the academic editor and reviewers. We hope the manuscript has been edited to satisfyingly address their concerns and the manuscript will now be suited for publication.

Sincerely,

On behalf of all authors,

Mari E. Deinhart

**Specific comments:**

**Please review your reference list to ensure that it is complete and correct. If you have cited papers that have been retracted, please include the rationale for doing so in the manuscript text, or remove these references and replace them with relevant current references. Any changes to the reference list should be mentioned in the rebuttal letter that accompanies your revised manuscript. If you need to cite a retracted article, indicate the article’s retracted status in the References list and also include a citation and full reference for the retraction notice.**

This Reference list in this manuscript revision has been changed. The order of citations has been changed because of the edits that occurred in this revision. Some additional references have been added (e.g., Mills et al., 2022) because we felt that were relevant to supporting an idea in this manuscript. There are a few references from the previous revision that were removed from this manuscript because they were not needed to show the purpose of this study.

**Reviewer 1: The authors have made great progress on this manuscript and improved the clarity, significance and addressed most of the issues raised by reviewers. However, some minor revisions are still needed on this manuscript. All the suggestions are explained in detail on an attached word document with track changes. The most significant area that needs work is the abstract, particularly the second half of the abstract where you talk about *Titanoderma*. There is no reference to how *Titanoderma* is related to any off the results you observed in the abstract and needs to be re-written. There are also several paragraphs throughout that are repetitive and/or not that relevant to the story being told. These paragraphs could be deleted, shortened or merged into other paragraphs.**

Thank you for taking the time to read our revisions and provide feedback to further improve this manuscript. The abstract, specifically the second half, has been edited per your suggestions (lines 15-39). We agree that the part discussing Titanoderma in the abstract did not fit with the message this manuscript wants to present so it was deleted. Repetitive paragraphs were removed from this revision, while other were merged into other paragraphs.

**Reviewer 2: Great job responding to the reviewers' suggested edits. The manuscript is significantly improved and the authors are to be commended for their detailed responses to reviewers' comments. On the attached are a few minor comments and edits.**

Thank you for taking the time to read through our manuscript and give suggestions to help improve it.

**Reviewer 1- Main Comments:**

**Reviewer 1: This whole abstract could use some work refining.**

Thank you for this suggestion, we refined the abstract to show the significance of this study (lines 15-39).

**Reviewer 1: I don’t think you need to state all the reasons why morphological identification are problematic in the abstract. I think a statement about what the consequences are of using morphological identifications would be more impactful. For example, results in lumping species together and not being able to accurately examine species-specific ecological functions.**

Thank you, these statements were deleted from the abstract and were replaced with “Most studies on the settlement preference of coral larvae on CCRA use morphological species identifications that can lead to unreliable species identification and do not allow for examining species-specific interactions between coral larvae and CCRA.” (Lines 18-21)

**Reviewer 1: I don’t think you can say that Lithophylloideae sp. 2 was significantly preferred as it was grouped with Lithophylloideae sp 3 & 4. Sentence needs rewording.**

That is a great point. Lithophylloideae sp. 2 was removed from this sentence, which was also reworded to say that Lithophylloideae sp. 1 was found to be ecologically important in this study (lines 30-34).

**Reviewer 1: This sentence doesn’t make sense in here, there is no reference to how Titanoderma is related to the species of CCRA that you observed preferential settlement on.**

Thank you, we agree and removed statements about *Titanoderma* from the abstract.

**Reviewer 1: Again, the identity of Titanoderma is a very minor component of the research and I don’t think it should be in the abstract. You can probably talk about previous research without mentioning Titanoderma. You could state, for example, that previous research on larval settlement preferences on other Acropora sp. have shown preferential settlement on a specific species of CCRA, however how this is related to the CCRA species you found to be important is unknown as these previous studies were based on morphological identifications.**

Thank you for the suggestion. Discussing *Titanoderma* here is not right. We have rephrased this section of the abstract (lines 34-39).

**Reviewer 1: Try to state why your findings of settlement preference are relevant! You could take the angle of driving the message of how the use of molecular methods makes you able to accurately assess settlement preferences and identify key CCRA that are important in the recovery of *A. surculose* populations that are seen as a dominate coral in healthy Guam coral reefs.**

Thank you very much for this suggestion. We rephrased the abstract to discuss how the use of DNA barcoding revealed species-specific roles and CCRA diversity that are often overlooked. This revised abstract also discusses how findings like this are beneficial for marine management and restoration efforts (lines 34-39).

**Reviewer 1: These are two different points, separate out into (1) and (2). You can reuse the same references.**

Thank you, these were separated out into (1) and (2). (Lines 53-54)

**Reviewer 1: I assume this is mainly algal overgrowth? Or is there overgrowth by other species? Perhaps state the species groups instead of just referring to them as nuisance species.**

Thank you for this suggestion. We kept your reference of ‘fleshy algae’ in this sentence (lines 56).

**Reviewer 1: Not sure you can state they are the most dominate species without a reference.**

Thank you, this sentence was reworded since it is based on our observations and there are not reports of the CCRA cover for Guam at this time (lines 56-58).

**Reviewer 1: This sentence repeats the above sentence. Especially the second half. Consider combining with sentence above. I think this whole paragraph can be deleted as it just repeats what is said above and doesn’t add a lot to the intro. Maybe some of the details could be integrated above.**

Thank you for pointing out the repetition of this paragraph from the previous one. The whole paragraph was removed from this manuscript.

**Reviewer 1: I don’t think this is needed (lines 87-90).**

Thank you, this was removed.

**Reviewer 1: I don’t think you should introduce this point about molecularly assisted identification until after you talked about why morphological identification are problematic in the paragraph below.**

Thank you, we kept your suggestions (line 88).

**Reviewer 1: State the amount of time (days or weeks) they were left in the tank before taking them out.**

Thank you, we included the number of days and the date the tiles were photographed for this study (lines141-143).

**Reviewer 1: Just state that it is bare tile or substrate.**

Thank you, this revision calls in bare tile (line 152).

**Reviewer 1: I think you need something here stating how the coralline recruits are counted. For example: “The number of coral recruits on each substrate category were visually counted during the sampling process and later confirmed during image analysis outlined below.” See comment on line 367 of results**

Thank you, your suggestions were added in (lines 155-160).

**Reviewer 1: This should be in the paragraph above and if you report the number of days or week the tiles were left (as suggested), then an exact date when photos were taken is not needed.**

Thank you. This sentence was moved up to the paragraph above (lines 142-143).

**Reviewer 1: You don’t need to explain a posthoc test. I think it is explained clearly enough above now.**

Thanks, the posthoc test explanation was removed.

**Reviewer 1: I don’t quite get the point of this paragraph and the need to explain what substrate category each of the five specimens that failed to sequenced fall into. From my understanding there were a number of specimens that were not collected for DNA analysis and were therefore visually assigned to one of your 8 substrate groups. Therefore, I don’t think you need an explanation on what they visually matched to, as this is exactly the same as how the CCRA samples that were not collected for sequencing were treated and you can probably delete the paragraph. Correct me if I am wrong? If this is not the case this paragraph is probably important.**

That is a great point, thank you. We removed the paragraph you are referring to.

**Reviewer 1: Very repetitive. Consider rewording into one sentence.**

Thank you for the suggestion. This paragraph was reworded to avoid repetition (lines317-319).

**Reviewer 1: I thought there were 9 categories. 8 CCRA and one bare tile. Check the correct number is stated throughout.**

You are correct, there were 9 categories. Thanks for pointing out this error.

**Reviewer 1: This is similar to what you wrote in the methods. I think instead of what you have here you need to make a statement about what you thought were individual species often represented multiple cryptic species, with the exception of two substrate categories (Lithophylloideae sp. 1 and Peyssonneliales sp. 5).**

Thank you for pointing that out. We have revised this paragraph and included a statement about the multiple cryptic species that morphological observation would not have been able to discern (lines 342-355).

**Reviewer 1: There was some minor pigmentation loss noted below. Adding the word “significant” deals with this issue.**

Thank you for making this suggestion that we kept.

**Reviewer 1: I think you can remove these sentences and just add in the methods that the coral recruits were counted using during the sampling process and confirmed during the image analysis.**

Thank you, we deleted these sentences from this paragraph and moved them to the materials and methods section (lines 156-160).

**Reviewer 1: Shouldn’t this say significant?**

Yes, this should say significant. Thank you.

**Reviewer 1: This paragraph needs some rearranging as it is very hard to follow, and some statements seem contradictory. Try rewording logically. The main points are 1) that there is a difference in cover of CCRA, 2) that the recruitment of coral onto these CRAA is different from random. For example, you explain the Lithophylloideae sp. 1 is the dominate species in three different areas in different ways, this could probably be all combined into one sentence. I made some suggestions but needs further work.**

Thank you for these helpful suggestions. This revised manuscript has followed your suggestion and rephrased the paragraph, so it is more concise and not repetitive (lines 374-382).

**Reviewer 1: I don’t see table 2 anywhere**

Thank you for pointing this out. There is no table 2 and this sentence was referring to S4 Table.

**Reviewer 1: This paragraph is a lot better! Try following a similar format when rewording the one above.**

Thank you.

**Reviewer 1: Because of the high number of species in this category is it likely that some species with a low cover could have actually been prime settlement substrate? Something to think about and maybe you could add a comment about this in the discussion?**

That is a great point, thank you! We address it in the discussion (lines 461-467).

**Reviewer 1: Add reference**

Thank you, a references were added (line 481).

**Reviewer 1: I wouldn't say that they are scarce. There are a large number of taxonomic studies on coralline algae and also a number of ecological studies. Yes, studies incorporating molecular taxonomy into ecological work are scarce, as most studies lump coralline algae or are based on taxonomy using only morphology. However, that is not what the sentence says as you currently have it worded.**

Thank you, that is a great suggestion. We have reworded this sentence to state that CCRA ecological studies using molecular taxonomy are less common (lines 481-483).

**Reviewer 1: Do you suspect that the tiles are representative of the natural community in the bay? My thoughts are you would find a larger number of species in the bay, particularly within a range of different micro-habitats and depths. You found very high diversity on the tiles, so likely even higher diversity in the bay. This is a significant point that you found large cryptic diversity on these artificial tiles that were housed in fixed environmental conditions. I think you should really emphases this and that you would likely find even higher diversity in the bay.**

Thank you for pointing that out. Yes, we have found that CCRA species richness for Pago Bay is much higher than what was found on the tiles. We refer to our accepted recently manuscript describing Guam’s CCRA diversity, which also discusses Pago Bay (lines 489-493).

**Reviewer 1: think I understand what you are trying to say but needs rewording to improve clarity. The fact that all the tiles were in the same tank suggest that environmental factors shape coralline communities. However, I am not sure you can state this, recruitment process could also drive communities (they were all recruited at the same time). There is also a lot of evidence of biotic factors including grazing influencing coralline communities Perhaps remove this sentence as I don't think it really adds to the discussion.**

That is an excellent suggestion, thank you. We rephrased the paragraph by removing that sentence and added in a sentence discussing how if tiles were exposed to differing abiotic factors, then the CCRA composition may vary (lines 534-538).

**Reviewer 1: Also, they were probably all exposed to the same algal spores which could differ if you added the tiles at a different timepoints.**

That is a great point. It is likely that the tiles were exposed to the same algal spore, we have no proof of it so we decided to not include this topic in there.

**Reviewer 1: What are you trying to convey with this statement? You do a good job in evaluating diversity and ecological value without having a well-characterised flora. I think this statement takes away value from the work you did in this study. I suggest rewording or removing.**

Thank you, we removed this statement.

**Reviewer 1: I think this section should go at the bottom of the discussion. It is important to know the identity of the species you are working with, but the main purpose of this study was to investigate settlement preferences.**

Thank you for the suggestion. We moved this to be the last section in the discussion (lines 539-561).

**Reviewer 1: This section is really good. I suggest you think about moving this section up to the start of the discussion as this is the most important aspect and what you set out to test in this paper. The other sections above still provide important information but were not the main purpose of the study.**

Thank you, we agree and have moved this section to be the first section in the discussion (lines 405-478).

**Reviewer 1: I think you need an extra sentence here stating how this is surprising it has not been detected on natural reefs given that it was the dominant species on your recruitment tiles.**

That is a good point, thanks. This revision has added a sentence here stating that it was surprising to have Lithophylloideae sp. 1 be a dominant species in this study since it has not been detected in Guam’s reef community to date (lines 431-435).

**Reviewer 1: If this section is moved up in the discussion, I would consider leaving this paragraph at the bottom as a conclusion.**

Thank you, we have made this paragraph the manuscripts conclusion (lines 563-575).

**Reviewer 2- Main Comments**

**Reviewer 2: Closely related is vague--what was the minimum value that you used?**

Thank you for pointing that out. Closely is a vague statement. We have rewritten this part of the paragraph to better describe what sequences were chosen to run the Peyssonneliales analysis (lines 244-255).
